# The State of the Art in Combination Locoregional and Systemic Treatment Strategies for Hepatocellular Carcinoma: Recent Advancements and Future Horizons

**DOI:** 10.3390/curroncol33030172

**Published:** 2026-03-17

**Authors:** Farbod Fazlollahi, Arianna D. Carfora, Marshal King, Elizabeth S. Wrasman, Mina S. Makary

**Affiliations:** 1 College of Medicine, The Ohio State University, Columbus, OH 43210, USA; 2 School of Medicine, Saint Louis University, St. Louis, MO 63103, USA; 3 Division of Vascular and Interventional Radiology, Department of Radiology, The Ohio State University Wexner Medical Center, Columbus, OH 43210, USA; 4 Department of Biomedical Engineering, The Ohio State University, Columbus, OH 43210, USA

**Keywords:** hepatocellular carcinoma, combination therapy, locoregional therapy, immunotherapy, transarterial chemoembolization, TACE, transarterial radioembolization, TARE, ablation, immune checkpoint inhibitors

## Abstract

Hepatocellular carcinoma remains one of the most common and lethal cancers worldwide, and many patients are diagnosed at stages where curative therapy is not possible. Recent progress in systemic therapies and refinements in locoregional treatment have shifted how clinicians approach this disease. As evidence has accumulated from trials such as KEYNOTE-937, IMbrave050, and CheckMate 9DX, it has become clear that pairing immunotherapy with ablation or transarterial interventions can deepen and extend treatment responses compared with using either approach alone. This review summarizes the current landscape of these combination strategies, explains the biological and clinical principles that support their use, and highlights ongoing trials that aim to clarify optimal sequencing and patient selection. It also considers future directions for integrating locoregional and systemic therapies to expand curative opportunities and improve long-term outcomes for a broader range of patients.

## Simple Summary

Liver cancer, specifically hepatocellular carcinoma, is one of the most common cancers and a leading cause of cancer death worldwide. Many patients are diagnosed at stages when surgery or liver transplant is no longer possible, leaving them with limited treatment options. Local tumor treatments, such as those that destroy cancer cells with heat or cold or those that cut off tumor blood supply, provide important alternatives for these patients. Numerous clinical trials have evaluated whether combining these local treatments with medications that boost the immune system or stop tumor growth can improve patient outcomes. This review examines progress made over the past two decades in developing these combination treatment approaches. Early evidence suggests that combining local and systemic treatments is generally safe and may help patients live longer, slow cancer progression, and in some cases reduce disease severity enough to make patients eligible for potentially curative surgery or transplant. However, questions remain about which combinations work best, when treatments should be given, and which patients benefit most. Focused discussion of completed and ongoing studies is essential to synthesize emerging evidence, inform optimal treatment strategies, and guide care in this rapidly evolving field.

## 1. Introduction

Hepatocellular carcinoma (HCC) remains a global public health challenge because of its rising incidence, diverse etiologic factors, and high mortality [1,2]. Clinical presentation varies widely, and prognosis depends not only on tumor burden but also on the degree of underlying liver dysfunction. Several staging systems are used to characterize disease, including the Barcelona Clinic Liver Cancer (BCLC), Okuda, Hong Kong Liver Cancer (HKLC), and Cancer of the Liver Italian Program (CLIP) classifications [3,4]. Among these, the BCLC framework is most widely applied because it links tumor stage and hepatic reserve to specific treatment strategies and expected survival outcomes (Table 1) [4].

Assessment of liver function is central to all staging approaches and the Child-Pugh score remains the standard measure of hepatic reserve (Table 1) [3,4]. Albumin, bilirubin, ascites, prothrombin time, and encephalopathy help determine treatment eligibility and influence decisions across the spectrum of locoregional and systemic therapy. Within the BCLC system, early-stage disease (BCLC 0 and A) is characterized by limited tumor burden and preserved liver function, and patients in this category may be candidates for surgical resection, transplantation, or ablative therapies [4,5,6]. Intermediate stage disease (BCLC B) typically involves multifocal tumors and is managed with transarterial therapies or, in select cases, systemic agents. Advanced stage disease (BCLC C) includes macrovascular invasion or extrahepatic spread and is treated primarily with systemic therapy. Terminal stage disease (BCLC D) reflects significant hepatic decompensation and poor functional status, for which supportive measures are recommended [6].

As HCC progresses, therapeutic options narrow, and the likelihood of achieving curative outcomes diminishes. This pattern has motivated interest in strategies that may extend benefit to patients who would otherwise be ineligible for curative treatment. Locoregional therapies (LRTs) and systemic agents have each demonstrated value across different disease stages and growing evidence suggests that their combination may enhance antitumor immune activation, improve progression-free survival (PFS), and preserve hepatic function [7,8]. These complementary mechanisms have prompted ongoing investigation into how best to integrate the two approaches as part of contemporary HCC management.

This review summarizes current advances in HCC treatment, with a focus on combination strategies that pair LRTs (including ablation, transarterial chemoembolization [TACE], and transarterial radioembolization [TARE]) with modern systemic and immunotherapeutic agents. It also highlights emerging evidence, ongoing clinical trials, and future directions that may further shape the role of combination therapy in HCC.

## 2. Methods

A comprehensive literature search was conducted using PubMed and Web of Science to identify studies published between January 2005 and December 2025. This range was selected to capture foundational work as well as contemporary developments in systemic, locoregional, and combination therapies for HCC. Clinical trials were identified through ClinicalTrials.gov, the EU Clinical Trials Register, and the European Organisation for Research and Treatment of Cancer database, among others. Search terms included “hepatocellular carcinoma”, “immunotherapy”, “systemic therapy”, “combination therapy”, “locoregional therapy”, “ablation”, “transarterial embolization”, “transarterial chemoembolization”, and “transarterial radioembolization”, and their identification numbers are provided in the text and associated tables. Reference lists of review articles were also examined for additional studies. Only English-language publications from peer-reviewed journals were included. Relevant data extracted from eligible studies included treatment techniques, clinical outcomes, adverse events, and conclusions. Trials without publicly available results were classified as “ongoing”.

## 3. Overview of Individual Therapeutic Modalities

Management of HCC relies on both systemic and locoregional therapies, each influencing tumor biology through distinct mechanisms. A brief overview of these modalities provides the clinical and mechanistic context needed to understand contemporary combination strategies.

### 3.1. Systemic Therapies

Systemic therapies for HCC include multikinase inhibitors (MKIs), immune checkpoint inhibitors (ICIs), and anti-angiogenic agents that target key pathways in tumor growth, angiogenesis, and immune evasion [9]. These drug classes underpin most completed and ongoing clinical trials evaluating combinations with LRTs.

#### 3.1.1. Multikinase Inhibitors

MKIs remain a cornerstone of systemic therapy for advanced HCC, particularly in patients presenting at BCLC stage C (Table 2) [1,4]. Sorafenib, the first systemic agent to demonstrate a survival advantage in HCC, inhibits RAF1, BRAF, VEGFR1-3, PDGFR, KIT, FLT3, RET, and FGFR1 [9,10,11]. Among these, VEGFR and RAF signaling are most relevant to HCC biology. In the SHARP trial (NCT00105443, Phase III, 178 international locations), sorafenib increased median overall survival (OS) from 7.9 to 10.7 months compared with placebo [10]. Real-world studies such as GIDEON (NCT00812175, Observational, 75 international locations) and INSIGHT (NCT03233360, Observational, 34 international locations) demonstrated similar outcomes in broader populations, including selected Child-Pugh B patients, although regulatory approval applies primarily to Child-Pugh A disease [10,12,13]. A specific advantage of MKIs is that their oral mode of treatment administration can help mitigate time and travel costs for patients who live far from cancer treatment institutions [14]. Common toxicities include palmar-plantar erythrodysesthesia, diarrhea, fatigue, hypertension, and gastrointestinal discomfort, and dose adjustment is frequently required.

Lenvatinib is a first-line alternative that inhibits VEGFR1-3, FGFR1-4, PDGFRα, KIT, and RET. In the REFLECT trial (NCT01761266, Phase III, 186 international locations), lenvatinib was noninferior to sorafenib and achieved a slightly longer median OS (13.6 months versus 12.3 months) [9,10,54]. Its adverse event profile reflects potent antiangiogenic activity, with hypertension, proteinuria, diarrhea, and hypothyroidism as characteristic toxicities [54].

Regorafenib serves as second-line therapy for patients who tolerate sorafenib but experience HCC progression. It inhibits VEGFR1-3, TIE2, RAF, KIT, RET, and FGFR, offering partially non-overlapping kinase inhibition that supports activity after sorafenib failure. The RESORCE trial (NCT01774344, Phase III, 147 international locations) demonstrated improved OS of 10.6 months with regorafenib compared to 7.8 months with placebo in HCC patients with Child-Pugh A liver function. Hypertension and hand-foot skin reaction are prominent toxicities, with diarrhea and fatigue appearing as class-consistent effects [55].

Cabozantinib inhibits MET, AXL, VEGFR2, and additional tyrosine kinases associated with tumor progression and resistance pathways. In the CELESTIAL trial (NCT01908426, Phase III, 104 international locations), cabozantinib improved median OS to 10.2 months compared with 8.0 months for placebo among previously treated patients with Child-Pugh A liver function [56]. A retrospective subgroup analysis of patients who progressed to Child-Pugh B status by week 8 reported median OS of 8.5 months with cabozantinib versus 3.8 months with placebo, suggesting tolerability and activity in patients with declining hepatic reserve [57]. Adverse events include diarrhea, decreased appetite, fatigue, hypertension, and palmar-plantar erythrodysesthesia.

It is worth noting that regorafenib and cabozantinib are currently used as first-line treatments following treatment with lenvatinib in the absence of phase 3 clinical trial data. Although immune-based combinations have shifted first-line therapy in many regions, MKIs remain highly relevant because of their efficacy, global accessibility, and compatibility with LRTs. Their antiproliferative and antiangiogenic effects, coupled with modulation of the tumor microenvironment and improved antigen release, provide a rationale for integrating MKIs into combination strategies.

#### 3.1.2. Immune Checkpoint Inhibitors

Immune checkpoint inhibitors have become an important component of systemic therapy for advanced HCC by restoring antitumor T-cell activity. Pembrolizumab and nivolumab are programmed cell death protein-1 (PD-1) inhibitors that block the interaction between PD-1 on T cells and PD-L1 expressed on tumor and immune cells, thereby preventing inhibitory signaling and enhancing cytotoxic T-cell responses [9,15]. In previously treated patients, pembrolizumab monotherapy achieved a median OS of 13.9 months in the KEYNOTE-240 trial (NCT02702401, Phase III), although the trial did not meet its pre-specified significance thresholds [16]. Nivolumab demonstrated durable responses in sorafenib-experienced patients in the CheckMate 040 trial (NCT01658878, Phase I/II, 60 international locations). However, nivolumab monotherapy did not improve OS compared with sorafenib in the Phase III CheckMate 459 trial (NCT02576509, 138 international locations), and its accelerated approval granted in 2017 was subsequently withdrawn in 2021 after negative confirmatory results [17,18,19]. PD-1 inhibitors commonly cause immune-related adverse events, including: rash, pruritus, diarrhea, colitis, hepatitis, and endocrinopathies [5,16].

Cytotoxic T-lymphocyte-associated protein-4 (CTLA-4) inhibition provides a complementary immunotherapeutic mechanism. Ipilimumab blocks CTLA-4, a key regulator of early T-cell priming in lymphoid tissues. In CheckMate 040, combination therapy with nivolumab and ipilimumab produced higher objective response rates and longer durations of response than PD-1 blockade alone in sorafenib-pretreated patients [17,18]. Immune-related toxicities were more frequent with combination treatment, but were generally manageable with standard immunosuppressive strategies [12]. CheckMate 9DW was a phase III trial that similarly employed ipilimumab-nivolumab combination therapy in patients who had no prior systemic therapy for HCC [20]. Median OS was higher in the combination therapy group compared to monotherapy with either lenvatinib or sorafenib (23.7 months vs. 20.6 months). Moreover, PFS and duration of treatment of response were especially favorable for combination therapy, and common adverse effects included rash, fatigue and diarrhea. The HIMALAYA trial evaluated sorafenib monotherapy and durvalumab monotherapy compared to combination therapy of tremelimumab and durvalumab which target CTLA-4 and PD-1, respectively. Although PFS was similar across arms, updated 4- and 5-year OS rates remain higher with combination therapy (4-year: 25.2%, 5-year: 19.6%). Common adverse effects include diarrhea, pruritic rash, fatigue, and abdominal and musculoskeletal pain [21,22,23].

Together, PD-1 and CTLA-4 inhibitors enhance T-cell activation at distinct points in the immune cascade. Their complementary mechanisms, capacity for durable responses, and compatibility with LRTs have positioned ICIs as central to evolving combination treatment strategies in HCC.

#### 3.1.3. Anti-VEGF/Angiogenesis-Targeted Therapies

Therapies targeting vascular endothelial growth factor (VEGF) signaling play an important role in HCC treatment by inhibiting angiogenesis and altering the tumor microenvironment. These agents either bind circulating VEGF ligands or block VEGF receptor activation on endothelial cells, thereby suppressing neovascularization and reducing tumor perfusion. Bevacizumab, a monoclonal antibody directed against VEGF-A, has demonstrated activity in early monotherapy studies but is primarily used in combination regimens because of its ability to modulate vascular permeability and enhance immune cell infiltration [24]. Characteristic adverse effects include hypertension, bleeding, and proteinuria, reflecting the dependence of normal vasculature on VEGF signaling. IMbrave150 demonstrated that VEGF/PD-1 treatment combination via bevacizumab and atezolizumab had an overall OS of 19.2 months compared to that of sorafenib monotherapy (13.4 months). Moreover, response rate was significantly elevated (29.8% vs. 5%) and common adverse reactions included hypertension, proteinuria, and fatigue [24].

Ramucirumab is a monoclonal antibody targeting VEGFR-2 and is approved for patients with advanced HCC with alpha-fetoprotein (AFP) levels of 400 ng/mL or higher. In the REACH-2 trial (NCT02435433, Phase III) and the AFP-high subgroup of the REACH trial (NCT01140347, Phase III), ramucirumab improved OS compared with placebo without significantly worsening quality of life [25,58]. Common toxicities include hypertension, hyponatremia, proteinuria, and increased AST and ALT levels [25].

VEGF pathway inhibition contributes not only to suppression of tumor angiogenesis but also to immunologic reprogramming within the tumor microenvironment. By reducing VEGF-mediated immunosuppression and improving T-cell infiltration, anti-VEGF therapies provide a mechanistic foundation for combination strategies involving ICIs and LRTs discussed later in this review.

### 3.2. Ablation Techniques

Ablative therapies remain a cornerstone treatment for patients with early-stage HCC who are not suitable candidates for surgical resection or liver transplantation. The primary goal is to induce complete tumor necrosis while preserving surrounding hepatic parenchyma. The most commonly used modalities include radiofrequency ablation (RFA), microwave ablation (MWA), and cryoablation (Table 2), each of which achieves cytotoxicity through distinct physical mechanisms [26].

#### 3.2.1. Procedural Overview

RFA is performed by inserting a percutaneous electrode into the tumor under imaging guidance. Delivery of alternating electrical current generates heat, resulting in coagulative necrosis within the tumor and a surrounding ablative margin. MWA delivers electromagnetic energy through one or more percutaneous antennae, producing rapid tissue heating that achieves cytotoxic temperatures and larger, more uniform ablation zones compared with RFA. Cryoablation induces cell death by cycling rapid freezing and thawing through compression and expansion of gases within cryoprobes, creating intracellular and extracellular ice crystals that disrupt membrane integrity and microvasculature. MWA and cryoablation can achieve larger ablative volumes with the simultaneous use of multiple probes.

Although the overall safety and oncologic outcomes of these techniques are broadly comparable, each modality has specific technical considerations. RFA is widely available, familiar to operators, and typically associated with shorter procedure times and rapid radiographic response. Its effectiveness, however, may be limited by the “heat-sink effect,” in which thermal energy dissipates into adjacent large vessels and prevents the tumor from reaching cytotoxic temperatures, leading to incomplete ablation or marginal recurrence. MWA is less susceptible to heat-sink effects because it generates higher intratumoral temperatures rapidly and can overwhelm cooling effects of local vasculature. It also often requires fewer probe insertions to achieve an adequate ablative margin, which may reduce procedural complexity. Cryoablation may be advantageous for small tumors abutting the diaphragm, bowel, gallbladder, or biliary tree, with potential for significantly lower complication rates compared with RFA for tumors near central biliary structures. Complications across modalities include bleeding, bile duct injury, abscess formation, and post-ablation syndrome; although, major adverse events remain uncommon [26].

#### 3.2.2. Clinical Considerations

Ablative therapies are indicated for patients with very early and early-stage HCC (BCLC 0/A) who demonstrate preserved liver function (Child-Pugh A and B7 without ascites or coagulopathy) and are not candidates for surgical resection or transplantation [6,59,60]. They are considered potentially curative in this population and are generally well tolerated. Treatment outcomes depend on several factors, most notably tumor size, with lesions under 5 cm demonstrating the greatest likelihood of complete ablation and reduced recurrence risk. Procedure planning must account for lesion location, as ablation in the hepatic hilum, subcapsular region, or immediately adjacent to the bowel, gallbladder, or major hepatic veins can increase the risk of thermal injury [61]. Contraindications include uncorrectable coagulopathy, uncontrolled ascites, decompensated liver function (Child-Pugh B8-9, C or higher), significant extrahepatic metastases, vascular invasion, and highly exophytic tumor growth [26]. Common adverse effects include post-procedural abdominal pain, bleeding, and thrombosis related to endothelial and parenchymal injury within the ablation zone [27]. Although infrequent, technique-specific complications may occur, such as biliary stricture or abscess formation. Cryoablation carries additional rare risks, including post-treatment “cryoshock” and tumor lysis syndrome [26,28,29].

Ablation offers a viable curative strategy for patients unable to undergo surgical intervention, particularly those with small, localized tumors [62,63]. A meta-analysis by Wang et al. demonstrated comparable 1- and 3-year OS between ablation and surgical resection for tumors under 5 cm, supporting ablation as a curative option in appropriately selected patients [30]. Among ablation modalities, survival outcomes are broadly equivalent. Several studies reported comparable 3-year OS rates when comparing RFA and cryoablation, and MWA achieves comparable 1-, 3-, and 5-year survival outcomes to RFA [31,32,33]. The practical importance of this equivalence is that it allows clinicians to select an ablation modality based on tumor location, heat-sink considerations, operator expertise, and procedural logistics rather than expectation of superior oncologic outcomes.

Evidence also supports a role for ablation in select intermediate-stage (BCLC B) patients. In a retrospective study by Ryu et al., MWA resulted in 1-, 3-, and 5-year OS rates of 98%, 74%, and 51%, respectively, with 1-year recurrence-free survival reaching 80% [64]. A separate 2024 study reported that 70% of patients with intermediate-stage disease demonstrated a radiographic response following multiple rounds of RFA during a 6-month observation period [65].

### 3.3. Transarterial Embolization

Transarterial embolization (TAE) is a foundational LRT for intermediate-stage HCC and underpins both transarterial chemoembolization (TACE) and transarterial radioembolization (TARE). The technique exploits the predominantly arterial blood supply of HCC to induce selective tumor ischemia while largely preserving normal hepatic parenchyma, which is primarily supplied by the portal vein (Table 2) [34,66]. Clinically, TAE is applied for disease control, symptom palliation, bridging to transplantation, and downstaging to curative therapies.

#### 3.3.1. Procedural Overview

Under advanced imaging guidance, TAE enables selective catheterization of tumor-feeding hepatic arterial branches, followed by delivery of embolic agents to achieve targeted flow reduction [39,67]. Commonly used materials include particulate embolics of varying sizes and compositions, while smaller particle sizes or liquid embolic agents such as Onyx, N-butyl cyanoacrylate, and ethanol may be selected when deeper penetration or rapid permanent occlusion is desired [34]. Temporary agents such as resorbable microspheres can be used when reversible occlusion is preferred. TACE and TARE represent extensions of this approach, combining embolization-induced ischemia with localized chemotherapy or internal radiation.

Technical success and safety are influenced by vascular anatomy and embolic selection [68]. Vessel tortuosity, small-caliber branches, and arterial stenosis can complicate catheter navigation and increase the risk of non-target embolization. Accordingly, embolic choice is tailored to tumor size, flow dynamics, and arterial anatomy, with particulate agents often used for larger tumors and high-flow feeders, and liquid embolics favored for small tumors or complex vascular patterns requiring durable occlusion. Unrecognized collateral arterial supply from sources such as the inferior phrenic, omental, or biliary branches may further limit treatment efficacy. Comprehensive angiographic mapping is therefore essential to achieve complete embolization while minimizing unintended ischemia [34,39,66,67].

#### 3.3.2. Clinical Considerations

TAE is an important therapeutic option for patients with BCLC A or B HCC who have adequate hepatic reserve and are not candidates for resection, ablation, or systemic therapy [5,34]. In selected patients, it may also serve as a bridge to liver transplantation [34,69]. Its clinical benefit is greatest in hypervascular tumors, which depend predominantly on hepatic arterial blood flow and are thus particularly amenable to embolization-based strategies [39]. Careful patient selection is critical, as contraindications include extensive portal vein thrombosis or vascular invasion, uncontrolled biliary obstruction, extrahepatic metastatic disease, severe renal dysfunction, and advanced cirrhosis with decompensated hepatic function [35,39]. When appropriately applied, TAE is generally considered a low bleeding risk procedure within interventional oncology [70].

Postembolization syndrome is the most common adverse event following TAE, manifesting as abdominal pain, nausea, vomiting, fatigue, and fever. Its incidence ranges from 60% to 90% and is influenced by baseline hepatic function and the degree of ischemic injury to non-tumoral parenchyma [36,37]. Although postembolization syndrome has been associated with shorter OS, this association likely reflects impaired hepatic reserve rather than a direct causal relationship [36,38]. Additional complications include biliary injury, hepatic abscess formation, pulmonary embolism, and progressive liver dysfunction, particularly in patients with marginal liver function prior to treatment [5,34].

Outcomes following TAE are strongly dependent on tumor burden, vascularity, and underlying liver function. A single-center series reported OS rates of 84.8% at 1 year, 38.3% at 3 years, and 18.7% at 5 years. TAE also enabled disease downstaging in approximately 14% of patients, facilitating access to additional therapeutic options [71]. Comparative studies suggest that OS is broadly similar among TAE, TACE, and TARE, though toxicity profiles differ [35,66]. While TAE is generally associated with fewer adverse effects than its chemoembolic and radioembolic counterparts, intrahepatic recurrence remains common and often necessitates repeat treatment or incorporation into combination therapeutic strategies [72].

### 3.4. Transarterial Chemoembolization

#### 3.4.1. Procedural Overview

Transarterial chemoembolization is the most widely used LRT for intermediate-stage HCC and is considered the standard of care for patients with BCLC B disease who retain adequate hepatic function. The technique builds on the principles of TAE by combining targeted delivery of chemotherapeutic agents with selective arterial occlusion, allowing high intratumoral drug concentrations while minimizing systemic exposure (Table 2) [39]. TACE is used for disease control, reduction of tumor burden, symptom management, and in certain cases, downstaging or bridging to curative therapies. The procedure uses the hepatic artery to deliver high concentrations of chemotherapeutic agents directly to the tumor while simultaneously restricting its blood supply. Two principal techniques are used in clinical practice: conventional TACE (c-TACE) and drug-eluting bead TACE (DEB-TACE) [39,72].

In c-TACE, chemotherapeutic agents such as doxorubicin, cisplatin, or 5-fluorouracil are mixed with iodized oil to create a water-in-oil emulsion that preferentially accumulates within hypervascular tumor tissue [12,39,72]. This is followed by administration of embolic particles to reduce washout, prolong local drug retention, and minimize systemic exposure [73]. The iodized oil component provides radiopacity, allowing visualization of treatment distribution on follow-up imaging, although systemic toxicity can be higher compared with other techniques [74].

DEB-TACE uses microspheres that are preloaded with chemotherapeutic agents. These beads lodge within tumor-feeding arterioles, where they provide mechanical embolization while releasing chemotherapy in a controlled, sustained fashion. This approach reduces systemic absorption, limits peak plasma concentrations, and standardizes drug delivery [39,72].

Alternative TACE methods include degradable starch microsphere TACE (DSM-TACE) and balloon-occluded TACE (b-TACE) [38,75]. DSM-TACE uses temporary embolic particles that dissolve after treatment, reducing the risk of prolonged ischemia to non-tumorous liver tissue. Balloon-occluded techniques employ an inflatable microcatheter to temporarily stop arterial inflow, permitting enhanced drug uptake within the tumor and lowering the likelihood of systemic toxicity. While these methods offer potential clinical advantages, they are less widely adopted than c-TACE and DEB-TACE.

#### 3.4.2. Clinical Considerations

TACE is widely regarded as the standard of care for most patients with intermediate-stage HCC who have preserved liver function and are appropriate candidates for embolization. It may also be used in select patients with very early or early-stage disease when resection or ablation is not feasible, when prior treatments have failed, or when downstaging or bridging to liver transplantation is required [44,76]. Contraindications include macrovascular invasion, significant portal vein thrombosis, uncontrolled biliary obstruction, and decompensated hepatic function [37,38]. Because TACE incorporates chemotherapeutic agents, it carries a higher risk of systemic adverse effects relative to bland embolization, including nausea, vomiting, and chemo-specific side-effects [39,69].

Clinical outcomes depend on tumor characteristics, hepatic reserve, and treatment response. Average response rates approach 50%, and one study reported 1-year OS exceeding 90% among patients with Child-Pugh A or B liver function [40,41,72]. Five-year survival in these cohorts typically ranged from 20 to 35% [40]. Tumor size influences prognosis, with markedly improved outcomes observed in lesions under 5 cm, where one cohort reported 1-year survival of 100% in Child-Pugh A patients and 94.1% in Child-Pugh B patients [40,41]. Additional prognostic factors include complete response to initial treatment and lower baseline AFP levels [42]. Although most commonly deployed in intermediate-stage disease, TACE has demonstrated efficacy in early-stage cohorts as well, with smaller studies reporting 1- and 3-year survival rates of 90.9% and 80.5%, respectively, and PFS nearing 26 months [43].

### 3.5. Transarterial Radioembolization

#### 3.5.1. Procedural Overview

Transarterial radioembolization is a catheter-directed therapy that delivers internal radiation to HCC lesions through selective infusion of radioactive microspheres into tumor-feeding branches of the hepatic artery (Table 2). Unlike TAE and TACE, TARE relies primarily on radiation-induced tumor necrosis rather than ischemia, which allows treatment of tumors that are poorly suited to embolic occlusion, including those with portal vein invasion or extensive collateral arterial flow [45]. The technique relies on deposition of high-energy particle emitters within the tumor microvasculature, where radioactive decay produces localized cytotoxic radiation with minimal ischemic effect on adjacent liver parenchyma. TARE is used for local tumor control, downstaging or bridging to liver transplantation, and treatment of advanced or multifocal disease in patients who maintain adequate hepatic reserve [77,78,79,80].

Two microsphere platforms are available in routine clinical practice: glass and resin. Glass microspheres contain higher radioactivity per sphere and are often selected for small or well-defined lesions requiring precise, high-dose delivery. Resin microspheres contain lower activity per particle but greater particle numbers, which may be advantageous for larger or more heterogeneous tumors. Both platforms require careful dosimetric planning to optimize tumoricidal effect while minimizing radiation injury to non-tumorous liver [45,77].

The most widely used isotope is yttrium-90, which emits high-energy beta particles with a tissue penetration range of approximately 2 to 10 mm, allowing treatment of both small and moderately sized lesions [45,81]. Holmium-166 is an emerging alternative radionuclide that emits both beta and gamma radiation, facilitating post-treatment imaging and allowing quantitative dosimetry. It has a shorter half-life than yttrium-90 and demonstrates similar safety and efficacy in early studies, although its use remains less widespread. Other isotopes investigated for radioembolization include rhenium-188, samarium-153, and iodine-131, but these are not commonly employed in contemporary practice [82,83,84].

#### 3.5.2. Clinical Considerations

TARE is used across a broad range of HCC stages and offers important advantages in specific clinical scenarios. In early-stage disease, TARE can be employed to slow progression in patients awaiting liver transplantation or to downstage tumors so that patients meet transplant eligibility criteria [46,85]. In intermediate-stage disease, TARE serves as an alternative to TACE, particularly for larger tumors or for patients who are not ideal candidates for chemotherapy-based embolization [81,86]. One of the distinctive strengths of TARE is its applicability in selected patients with advanced disease. Because radioembolization relies primarily on radiation rather than ischemia, it can be used safely in patients with portal vein invasion, a setting in which other embolic techniques are typically contraindicated [87]. General contraindications include extrahepatic metastatic disease, significant biliary obstruction, and decompensated hepatic function [39,72]. It is worth noting that retrospective comparisons indicate that TARE is preferred to TACE in the setting of limited portal vein and/or hepatic vein thrombus because risk of arterial occlusion is lower in TARE [88].

As with other transarterial treatments, TARE carries risks related to arterial catheterization and embolic delivery, but it also requires awareness of radiation-specific complications [36,87]. Radioembolization-induced liver disease is characterized by jaundice and ascites occurring 1–2 months after treatment and has an incidence below 10% [89,90]. Rarely, shunting of microspheres to the pulmonary circulation may precipitate radiation pneumonitis, which typically presents approximately 3 months after treatment with dyspnea, chest discomfort, and, in severe cases, progressive fibrosis [52,53]. The incidence of clinically significant pneumonitis is under 1% when standard preprocedural lung shunt evaluation is performed [91].

TARE has demonstrated promising efficacy across clinical stages. Reported response rates range from approximately 77% to 90% [47,92]. A multicenter analysis found median OS of 24 months for BCLC A patients, 17 months for BCLC B patients, and 10 months for BCLC C patients [48]. A more recent retrospective analysis showed a 3-year OS rate of 87% across treated patients and a 93% survival rate among those receiving TARE as a neoadjuvant therapy before resection or transplant [49]. When compared with TACE, multiple studies report similar OS, but consistently longer time to progression and lower toxicity with TARE [50,51,67]. Similar to its counterparts, TARE treatment response is influenced by baseline tumor stage, tumor size and burden, liver function, and the presence of extrahepatic disease [93].

## 4. Combination of Therapeutic Strategies

Combination approaches in HCC have gained prominence as advances in interventional oncology and systemic therapy reveal complementary strengths across modalities. Locoregional treatments can achieve rapid cytoreduction and promote immunogenic tumor injury while systemic agents provide broader disease control and suppress mechanisms of resistance that limit local therapy durability. As evidence accumulates from mechanistic studies and clinical trials, a clearer picture is emerging of how best to integrate these modalities to improve outcomes across disease stages.

### 4.1. Mechanistic Rationale

The rationale for combining systemic therapies and LRTs in HCC emerges from a growing understanding of how these treatments interact with tumor biology, hepatic architecture, and the immune microenvironment. Locoregional therapies generate controlled tumor injury through thermal destruction, ischemia, or internal radiation, which produce immediate cytotoxic effects and trigger release of tumor-associated antigens and cytokines into the surrounding environment. This antigen-rich environment, supported by dendritic cell activation and transient inflammatory recruitment, creates a biologic window in which antitumor immunity can be meaningfully amplified [94,95,96]. This endogenous response, however, is typically short lived. Tumors rapidly deploy counter-regulatory mechanisms (including upregulation of PD-1 and its ligand, PD-L1), mobilize myeloid-derived suppressor cells, and re-establish an immunosuppressive cytokine milieu. These adaptations help explain the high rates of local recurrence following locoregional monotherapy [15].

Systemic agents offer complementary mechanisms that may stabilize or enhance the immune activation initiated by local treatments. Immune checkpoint inhibitors mitigate the rebound inhibitory signaling that follows ablation or embolization and sustain activation of cytotoxic T-cells against residual microscopic disease. Multikinase inhibitors targeting pathways such as VEGF, fibroblast growth factor, and platelet-derived growth factor (PDGF) alter tumor vascularity, reduce angiogenic signaling, and remodel the tumor microenvironment. These effects can improve anti-tumoral immune cell trafficking and reduce heat-sink limitations during ablation. Antiangiogenic therapy may also normalize aberrant vasculature transiently, improving delivery of immunotherapeutic agents and facilitating deeper penetration of inflammatory cells.

Beyond immunologic synergy, combination approaches address limitations inherent to each modality. Locoregional therapies are highly effective at cytoreduction but do not reliably eradicate microscopic vascular invasion or distant intrahepatic foci. Systemic therapies provide broader disease control but often lack the capacity for rapid tumor debulking. Integrating the two allows immediate cytotoxic clearance with durable systemic immune pressure. This strategy is particularly relevant for a disease characterized by multifocality, vascular invasion, and an underlying cirrhotic substrate that restricts both therapeutic choices and tolerability. Such a mechanistic framework underlies the expanding landscape of clinical trials designed to define optimal treatment combinations, sequences, and patient selection criteria across the full spectrum of HCC therapy.

### 4.2. Ablation Combined with Systemic Therapy

Ablation exemplifies the pattern described above: rapid cytotoxic injury releases tumor-associated antigens and cytokines, yet recurrence rates after monotherapy suggest insufficient immune-mediated disease control. Ablation-specific features further strengthen the rationale for combination therapy. Thermal techniques are susceptible to heat-sink effects from adjacent vessels, which can limit complete tumor destruction. Antiangiogenic agents may reduce these effects by modifying tumor perfusion, leading to more uniform thermal coagulative necrosis and improved local control [41]. Preclinical studies also demonstrate enhanced susceptibility to PD-1 inhibition when vascular and immunosuppressive pathways are concurrently suppressed by MKIs such as sunitinib [97]. These mechanistic synergies support ongoing clinical trials evaluating ablation in combination with immunotherapy and targeted therapy, with the aim of strengthening both immediate cytotoxic effects and long-term systemic immunity.

#### 4.2.1. Completed Studies

Radiofrequency ablation has been evaluated in combination with multiple systemic agents over the past decade, with early studies focusing primarily on MKIs (Table 3). Trials conducted by Gong et al., Fukuda et al., and Feng et al. examined the addition of sorafenib in patients who were largely early stage HCC (BCLC A) with mean tumor sizes near 3 cm [98,99,100]. Treatment sequencing varied across studies. Gong et al. initiated sorafenib 28 days after ablation, Fukuda et al. administered sorafenib 7 days prior to ablation, and Feng et al. administered sorafenib either 60 days before or after ablation [101]. Despite differences in timing, all three studies reported synergistic improvement in PFS or OS (Table 3). Gong et al. additionally observed reduced relapse and prolonged tumor-free survival with delayed post-ablation sorafenib initiation. A study by Kan and colleagues evaluated sorafenib combined with ablation for tumors larger than 3 cm and reported a median PFS of 17 months [102]. Adverse effects across these trials reflected expected risks of ablation and sorafenib, including: bleeding, ascites, procedure-related injury, and dermatologic or gastrointestinal toxicities. Subsequent studies have explored alternative MKI partners. In a 2022 cohort, Wang et al. reported that lenvatinib combined with ablation improved response durability, PFS, and OS in patients with a median tumor size of 4.77 cm. Hypertension, elevated transaminases, and gastrointestinal intolerance were the most frequent toxicities [103].

Immune checkpoint inhibitors have also been studied in the periablational setting. The NIVOLVE Phase II trial (UMIN 000026648, Japan) evaluated adjuvant nivolumab after ablation or surgical resection and demonstrated a recurrence-free survival of 26.3 months, with no significant difference between the two approaches. Recurrence risk correlated with reduced CD8-positive T-cell infiltration and activation of the WNT or beta-catenin pathway [104]. Additional studies combining ablation with nivolumab or pembrolizumab reported increases in objective response rate from 10% with monotherapy to 24% with combined treatment [105]. Trials incorporating PD-1 inhibitors, such as toripalimab, camrelizumab, and sintilimab, in recurrent Child-Pugh A HCC have demonstrated improved efficacy with acceptable safety profiles [106,107,108].

Early work has also examined CTLA-4 inhibition combined with ablation. A single-arm study by Duffy et al. (NCT01853618, USA) evaluated neoadjuvant tremelimumab with ablation (cryoablation or RFA) or TACE and reported a median OS of 12.3 months, without dose-limiting toxicities. Pruritus was the most common adverse effect. Although this study did not include an ablation monotherapy comparison arm, it demonstrated that tremelimumab produced similar outcomes when combined with either ablation or TACE [109]. Subsequent trials have not yet expanded on CTLA-4 blockade paired specifically with ablation.

Collectively, these completed studies illustrate the breadth of systemic agents that may enhance the immunologic and oncologic effects of ablation. The consistency of improved outcomes across MKIs and ICIs highlights the potential for ablation-based combination strategies and informs ongoing efforts to refine optimal timing, sequencing, and patient selection.

#### 4.2.2. Ongoing Studies

Several Phase II and Phase III trials are actively evaluating ablation in combination with immune checkpoint blockade or targeted systemic agents (Table 4). These studies are designed to clarify optimal sequencing, identify candidate immunotherapies for periablational use, and assess whether synergistic responses observed in earlier trials translate to improved long-term outcomes in broader patient populations.

A major focus of ongoing research involves evaluating benefits of immunotherapy in patients who are LRT-naive. Multiple Phase II trials follow this framework. The NIVOLEP trial (NCT03630640, France), completed in 2023, investigated the neoadjuvant versus adjuvant use of nivolumab combined with curative intent tumor electroporation, although results have not yet been reported. Another Phase II study (NCT04652440, China) is assessing safety and tolerability of tislelizumab (PD-1 inhibitor) delivered before and after RFA or MWA. Additional PD-1 inhibitors are being investigated in similar designs. NCT04663035 (China) is evaluating the efficacy of CT-guided RFA with or without adjuvant tislelizumab, while NCT04150744 (China) is measuring PFS with camrelizumab monotherapy versus RFA combination therapy. These designs may help delineate the relative contributions of immunotherapy and locoregional treatment.

Several large Phase III studies are expected to report within the next 5 years. These trials typically enroll patients with preserved hepatic function who have undergone resection or ablation prior to randomization. CheckMate 9DX (NCT03383458, 184 international locations) is evaluating nivolumab versus placebo as adjuvant therapy to prevent post-resection or post-ablational recurrence. KEYNOTE-937 (NCT03867084, 249 international locations) compares pembrolizumab with placebo after complete resection or ablation, and IMbrave050 (NCT04102098, 134 international locations) is assessing whether combined atezolizumab (PD-L1 inhibitor) and bevacizumab versus active surveillance improves recurrence-free outcomes after complete resection or ablation [24]. The focus of these trials on recurrence prevention is particularly relevant, as they address whether systemic immunotherapy can reduce recurrence risk and extend the durability of curative-intent locoregional treatment.

Collectively, ongoing Phase II and III trials are expected to define the role of periablational immunotherapy more clearly, establish comparative benefits among available agents, and inform optimal sequencing strategies for integrating ablation with modern systemic treatments.

### 4.3. Embolization Combined with Systemic Therapy

Embolization-based therapies induce ischemic tumor injury that enhances antigen release, disrupts immunosuppressive signaling, and alters tumor vascular dynamics in ways that can potentiate the effects of systemic treatment [110]. Hypoxia generated by arterial occlusion increases expression of PD-L1 and hypoxia-inducible factor 1α, both of which promote immune escape and stimulate rebound angiogenesis [111,112,113]. These adaptations provide a strong rationale for combining embolization with systemic agents that inhibit VEGF or PDGF signaling. In addition, ischemia increases cell membrane permeability and diminishes efflux pump activity, thereby improving intratumoral accumulation of chemotherapeutic or targeted agents administered systemically [114]. Collectively, these ischemic and immunologic effects form the mechanistic basis for evaluating TAE and TACE in combination strategies that aim to strengthen both local tumor control and systemic antitumor activity.

#### 4.3.1. Completed TACE Studies

**Single-agent systemic combinations.** Transarterial chemoembolization is a standard therapy for intermediate stage HCC, but recurrence remains common, prompting evaluation of combination strategies incorporating systemic agents [72]. Several trials have tested the addition of sorafenib to TACE, including TACTICS (NCT01217034, Japan), TACE-2 (EudraCT, number 2008-005073-36, and ISRCTN, number ISRCTN93375053, UK), SPACE (NCT00855218, 107 international centers), and POST-TACE (NCT00494299, 75 locations Japan and Korea) (Table 5) [115,116,117,118]. These studies generally enrolled patients with preserved hepatic function, Child-Pugh Class A status, and adequate baseline performance, while excluding those with extrahepatic spread, extensive thrombosis, excessive tumor burden, or contraindications to TACE [115,116,117,118].

Although all four trials evaluated similar sorafenib doses, treatment sequencing varied substantially. POST-TACE initiated sorafenib after embolization, whereas other trials administered sorafenib before the procedure [115]. Adverse effects reflected expected toxicities of sorafenib and TACE, including postembolization syndrome, hand-foot skin reaction, fatigue, diarrhea, and nausea [115,116,117,118].

Results across these trials have been heterogeneous. TACTICS demonstrated the most favorable outcomes, including a PFS of 22.8 months compared with 13.5 months in the control group and an OS difference of at least 6 months (Table 5). By contrast, SPACE and TACE-2 showed minimal differences between sorafenib and placebo, and POST-TACE did not demonstrate a survival benefit. Collectively, these studies did not produce uniform evidence supporting sorafenib-augmented TACE [115,116,117,118]. However, their divergent findings helped clarify endpoint selection, highlighted the challenges of standardizing TACE protocols across centers, and informed the design of subsequent trials [119].

Research has since expanded to other systemic partners. The Phase II TACTICS-L trial (jRCTs031180074, Japan), completed in 2023, evaluated lenvatinib administered before and after TACE using eligibility criteria similar to earlier studies [120]. Lenvatinib achieved a median PFS of 28 months, meeting its primary endpoint, although OS did not differ significantly [120]. The Phase III LAUNCH trial (NCT03905967, China) further demonstrated improved PFS and OS with lenvatinib plus TACE compared with lenvatinib alone [121]. These findings are clinically relevant because TACE-mediated tumor debulking can improve hepatic function and enhance the effectiveness of lenvatinib, facilitating downstaging in selected intermediate stage patients [121].

Immunotherapy has also been paired with TACE in completed trials. IMMUTACE (NCT03572582, Germany), a Phase II study evaluating biweekly nivolumab following an initial round of TACE, enrolled patients with intermediate stage HCC, including some with prior resection or radiotherapy. This trial reported a median OS of 28.3 months and a PFS of 7.2 months, with a response rate of 74.1%, including a partial response in 55.1% of patients. Common adverse effects included fatigue and transient elevations in liver enzymes [122].

**Table 5:** Summary of completed TACE/immunotherapy studies.

Trial	Status	Treatment	Study Size	Population Characteristics	OS (or RFSR)	PFS (or RFS)	Most Common Adverse Effects	Did Combinatorial Treatment Outperform Monotherapy?	Additional Details
POST-TACE [115]	Completed (Phase III)	Sorafenib + TACE	458	Child-Pugh A	29.7 months	5.4 months	Postembolization syndromes, fatigue, hand-foot syndrome, nausea, vomiting, diarrhea	Yes	5.4 months TTP
TACTICS [116]	Completed		156	Child-Pugh A	36.2 months	22.8 months		Yes	Compared combination to TACE alone
TACE 2 [117]	Completed (Phase III)		313	Child-Pugh A	21.1 months	7.8 months		No significant differences	10.7 months TTP
SPACE [118]	Completed (Phase II)		307	BCLC B; Child-Pugh A	18.2 months	n/a		No significant differences	5.5 months TTP (vs. 5.4 months placebo)
TACTICS-L [120]	Completed (Phase II)	Lenvatinib + TACE	62	BCLC 0-B; Child-Pugh A; unresectable HCC, no previous systemic therapy	Not reached	28.0 months	Hypothyroidism, fatigue, hypertension, proteinuria, abdominal pain	Single arm	n/a
LAUNCH [121]	Completed (Phase III)		336	BCLC 0-B; Child-Pugh A; no previous treatment	17.8 months	10.6 months		Yes	n/a
IMMUTACE [122]	Completed (Phase II)	Nivolumab + TACE	59	BCLC B; Child-Pugh A	28.3 months	7.2 months	Elevated liver enzymes	Single Arm	n/a
PETAL [123]	Completed (Phase Ib)	Pembrolizumab + TACE	15	Child-Pugh A	33.5 months	8.95 months	Fatigue, weight loss, rash	Single Arm	n/a
Guo et al. [124]	Completed (Phase II)	Sintilimab + TACE	60	BCLC A-B; Child-Pugh A	n/a	30.5 months	Elevated liver enzymes, decreased albumin, anemia, weight loss, fatigue	Single Arm	51 patients received surgical resection after combination therapy, did not meet PFS
Checkmate 74W [125]	Completed (Phase III)	(Nivolumab ± Ipilimumab) + TACE	26	BCLC B	n/a	n/a	Pruritic rash, fatigue, infection, diarrhea, pyrexia, pain	n/a	Overall occurrence of adverse effects was similar in all 3 groups; serious adverse effects were observed in higher rates in combination treatment groups

The PETAL Phase Ib trial (NCT03397654, UK) investigated pembrolizumab administered every 3 weeks following two sequential rounds of TACE [123]. Median PFS was 8.9 months and OS was 33.5 months. Although 93% of patients experienced treatment-related adverse effects, most were manageable, and no synergistic or dose-limiting toxicities were observed. Additional work with sintilimab combined with TACE demonstrated a preoperative response rate of 62%, with 51/60 patients able to proceed to surgical resection. Median PFS was 30.5 months, and the endpoint was not reached in patients who ultimately underwent surgery [124].

Together, these completed studies demonstrate that TACE can be safely combined with MKIs or ICIs and that certain combinations, particularly lenvatinib-based strategies, may improve clinical outcomes in selected patients. At the same time, the mixed results from early sorafenib trials highlight the importance of standardizing TACE protocols, optimizing drug sequencing, and refining trial endpoints to better assess synergy in future investigations.

**Multi-agent systemic combinations.** Several contemporary studies have evaluated TACE in combination with systemic regimens that incorporate more than one therapeutic class, including dual immune checkpoint blockade and combined immunotherapy with antiangiogenic agents. These trials reflect a growing interest in strategies that leverage complementary mechanisms to enhance the immunologic and cytotoxic effects of TACE.

CheckMate 74W (NCT04340193, 107 international locations) is a randomized, multi-center, double-blinded, placebo-controlled Phase III study comparing nivolumab and ipilimumab, nivolumab monotherapy, and placebo combined with TACE in intermediate stage HCC [125]. Preliminary data indicate that adverse events have been comparable across treatment groups, although 3/9 patients receiving nivolumab and ipilimumab required discontinuation because of treatment-related toxicity. Efficacy results are anticipated following completion of follow-up, and this study represents one of the earliest efforts to pair TACE with dual checkpoint inhibition.

These trials demonstrate the feasibility of integrating TACE with multi-class systemic agents and highlight the potential advantages of engaging multiple immune and angiogenic pathways simultaneously. As results mature, these studies are expected to clarify whether combined immunotherapy and antiangiogenic therapy can meaningfully enhance the clinical benefits of TACE in intermediate stage HCC.

#### 4.3.2. Ongoing TACE Studies

**Single-agent systemic combinations.** Several ongoing trials continue to evaluate TACE in combination with single-class systemic agents, particularly PD-1 inhibitors, in order to clarify optimal sequencing, dosing schedules, and patient selection for combination strategies (Table 6). A recently completed study (NCT04297280) examined sintilimab administered every 3 weeks following TACE in patients with BCLC B or C HCC. Its design paralleled that of IMMUTACE, using post-TACE PD-1 blockade to enhance immune activation after LRT. Results have not yet been reported [122].

Additional early-phase investigations include an Early Phase I study of DEB-TACE combined with nivolumab in HCC patients (NCT03143270, USA), expected to complete in spring 2026. This trial incorporates three dosing cohorts to explore timing effects: nivolumab biweekly starting two weeks after DEB-TACE; nivolumab biweekly starting four weeks prior to, but not on the day of DEB-TACE; nivolumab biweekly starting four weeks prior to and on the day of DEB-TACE. All cohorts will receive nivolumab for one year following nivolumab biweekly starting four weeks prior, but not on the day of DEB-TACE. These cohorts are designed to study potential dose-related toxicity, after which future phases would be poised to determine the effectiveness of the combination of embolization with drug-eluting beads and systemic immunotherapy in advanced HCC.

TACE-3 (NCT04268888, France and UK) is a Phase II/III study evaluating nivolumab and embolization in intermediate HCC not amenable to surgery. This trial is notable for comparing TACE or TAE monotherapy to combination therapy with systemic PD-1 inhibition. The design aims to determine whether PD-1 inhibition following embolization improves PFS or OS compared with embolization alone and whether outcomes differ between chemoembolization and bland embolization when paired with immunotherapy. These ongoing trials are expected to generate important insights regarding the timing, dosing, and clinical benefit of pairing TACE with single-class systemic therapies, particularly ICIs.

**Multi-agent systemic combinations.** Ongoing trials are increasingly focused on evaluating TACE in combination with regimens that incorporate more than one systemic therapeutic class, including dual immune checkpoint inhibition or combined ICI/MKI therapy with antiangiogenic agents. These studies aim to clarify whether engaging multiple immune or vascular pathways can further potentiate the effects of TACE in intermediate or advanced HCC.

The EMERALD-1 Phase III trial (NCT03778957, 166 international locations) represents a major advance in evaluating combined immunotherapy and antiangiogenic therapy in the setting of TACE. This study is investigating the combination of TACE with durvalumab, a PD-L1 inhibitor approved in 2022, with durvalumab and bevacizumab, or with placebo and demonstrated improved PFS and acceptable safety profiles for the dual drug combination regimen [126]. Overall survival follow-up is ongoing. Durvalumab is also being evaluated in the related EMERALD-2 study (NCT03847428, 217 international locations), which is investigating its use as adjuvant therapy in patients treated with resection or ablation, and the EMERALD-3 study (NCT05301842, 171 international locations), which is investigating the concurrent treatment with durvalumab, tremelimumab, and TACE with or without lenvatinib compared with TACE monotherapy [127,128]. Whereas EMERALD-1 enrolled patients with unresectable HCC, EMERALD-2 includes individuals who have already undergone curative-intent LRT or resection and remain at high risk for recurrence, and EMERALD-3 includes individuals with locoregional HCC who are not candidates for curative-intent surgery or ablation. These designs will help determine the differential efficacy of durvalumab, alone or in combination with other agents, can reduce recurrence in patients treated with curative intent LRTs.

TALENTACE (NCT04712643, China and Japan) is another important ongoing randomized open-label Phase III study evaluating TACE with combined atezolizumab and bevacizumab in treatment-naive Child-Pugh Class A HCC patients. Unlike more rigidly timed protocols, TALENTACE focuses on patients at high risk for poor post-TACE outcome and allows TACE to be performed as clinically indicated rather than according to a fixed schedule. This flexible design may provide insight into how the timing of embolization relative to immune checkpoint inhibition and antiangiogenic therapy influences treatment efficacy. The estimated completion year is 2029.

LEAP-012 (NCT04246177, 205 international locations) is a Phase III randomized, double-blind, placebo-controlled trial that is comparing lenvatinib plus pembrolizumab combined with TACE versus TACE with matched placebos in patients with unresectable, non-metastatic HCC. Inclusion criteria targeted patients with Eastern Cooperative Oncology Group performance status of 0 or 1, Child-Pugh class A liver function, and liver-confined multinodular disease, including those with early-stage tumors unsuitable for curative-intent therapies. This is the first Phase III trial to compare locoregional therapy with immune-based systemic combination therapy in this population. Interim results demonstrated superiority in PFS (14.6 vs. 10.0 months by RECIST 1.1; HR 0.66) with a trend toward improved OS (HR 0.80; *p* = 0.087) [129].

Another ongoing study (NCT05608200, China) is evaluating lenvatinib and TACE versus lenvatinib, TACE, and sintilimab in patients with BCLC C disease. This trial will help clarify whether adding PD-1 inhibition confers additional benefit when combined with MKI-aided TACE in advanced-stage HCC.

Another active investigation (NCT04992143, China) is assessing TACE combined with tislelizumab followed by sorafenib in BCLC C patients. The sequential design, in which TACE is followed by PD-1 inhibition and subsequently an MKI, may offer some insight into whether treatment order meaningfully affects clinical outcomes. Although the trial was completed in 2023, results have not yet been reported.

Collectively, these studies reflect ongoing interest in identifying synergistic multi-class systemic strategies that enhance the therapeutic effect of TACE. Their results are expected to clarify the contribution of treatment timing, sequencing, and multimodal pathway targeting to clinical outcomes in intermediate and advanced HCC.

#### 4.3.3. Completed TARE Studies

Of the established LRTs for HCC, TARE is the most recently developed, and fewer completed trials have evaluated its performance in combination with systemic therapy (Table 7) [93]. Early investigations primarily examined the integration of MKIs. SORAMIC (NCT01126645, Phase II, Germany), SARAH (NCT01482442, Phase III, 26 locations, France), and SIRveNIB (NCT01135056, Phase III, 29 international locations) are among the largest studies completed to date and enrolled patients with intermediate or advanced unresectable HCC between 2010 and 2016. Although these trials differed in design, they collectively assessed whether incorporating TARE into systemic therapy improves outcomes beyond sorafenib monotherapy [130,131,132].

SORAMIC was the only major study in this group to directly evaluate TARE combined with sorafenib, with inclusion criteria requiring no prior systemic therapy. In contrast, SARAH and SIRveNIB compared TARE alone to sorafenib alone. SARAH required either ineligibility for surgery/LRT or recurrence after surgery/LRT, whereas SIRveNIB more broadly included patients who were either ineligible for or would be sub-optimally managed with ablation alone. Exclusion criteria across these studies were consistent with other combination-therapy trials and included extrahepatic metastases, significant prior resections, and contraindications to angiographic procedures [72,130,131,132].

Across trials, median OS was similar between treatment groups, with pooled average median OS values of 11.3 months. SARAH and SIRveNIB reported slightly longer OS of nearly 2 months in sorafenib-only groups, although none of the trials demonstrated statistically significant differences in OS (Table 7) [130,131]. Importantly, TARE was associated with lower rates of high-grade adverse events and improved patient-reported quality of life across multiple domains. These findings suggest that while survival outcomes may be comparable, the tolerability and symptomatic benefit of TARE warrant continued evaluation of combination or sequential approaches.

Additional completed studies have focused on pairing TARE with nivolumab. The NIVO Phase I study (NCT02837029, USA) administered low-dose (80 mg) or high-dose nivolumab (240 mg) following TARE in patients with advanced-stage HCC and established a recommended dose while demonstrating an early clinical benefit rate of 82% [133]. NASIR-HCC (NCT03380130, Phase II, Spain) evaluated nivolumab initiated 3 weeks after TARE in Child-Pugh A patients with BCLC B disease [134]. Median OS reached 20.9 months with a median time to progression of 8.8 months, with 4/42 patients successfully downstaged to resection. Pembrolizumab has also been explored following TARE in a cohort including Child-Pugh A and B patients (NCT03099564, Early Phase I, USA). This study reported a median OS of 27.3 months and PFS of 9.9 months, indicating the potential feasibility of combination strategies even in patients with impaired hepatic reserve [135].

Collectively, these completed studies highlight the growing interest in pairing TARE with targeted therapies and ICIs. While survival benefits remain modest in early trials, consistent improvements in tolerability, quality of life, and downstaging rates demonstrate the need for continued exploration of TARE-based combination strategies.

#### 4.3.4. Ongoing TARE Studies

As with other modalities of LRTs, PD-1 inhibition remains a central focus of current investigations evaluating radioembolization in combination with systemic therapy (Table 8). A Phase II study (NCT03033446, Phase II, Singapore), estimated to complete in 2025, is examining the efficacy and safety of nivolumab administered 3 weeks after TARE. This trial will help clarify whether sequential introduction of immunotherapy after radioembolization enhances disease control in patients with preserved liver function.

Several trials are also exploring combined PD-1 and CTLA-4 blockade with durvalumab and tremelimumab. Multiple studies within this category are projected for completion in 2025. A Phase Ib study (NCT04605731, USA) is evaluating post-TARE immunotherapy in patients with BCLC B and C disease, providing early safety and feasibility data in locally advanced HCC populations. The IMMUWIN trial (NCT04522544, Phase II, Germany) is comparing immunotherapy after TARE versus DEB-TACE, a design that may help identify scenarios in which different embolization modalities are preferentially suited for combination strategies. The ROWAN trial (NCT05063565, Phase II, 36 international locations) is a similar investigation that evaluates identical agents but employs a different dosing schedule, using a single priming dose of tremelimumab paired with monthly durvalumab administration for 18 months. ROWAN is positioned to clarify how dosing and treatment sequencing influence safety, durability, and overall response.

Additional efforts include EMERALD-Y90 (NCT06040099, USA), a Phase II study evaluating durvalumab combined with bevacizumab following TARE, with estimated completion in 2026. This trial extends the ongoing investigation of antiangiogenic and immune checkpoint synergy into the setting of internal radiation therapy. Interest in combined VEGF and PD-1 inhibition remains high, despite the termination of a prior trial (NCT04541173, Phase II, USA) due to slow accrual. Results from ongoing studies are expected to define optimal timing, sequencing, and patient selection for TARE-based combination therapy.

## 5. Discussion

The convergence of systemic and LRTs in HCC reflects a fundamental shift in how the disease is conceptualized and treated. Where monotherapies once dominated clinical practice, emerging mechanistic insights and expanding clinical trial data now suggest that durable control of HCC often requires simultaneous cytoreduction, modification of the tumor microenvironment, and sustained systemic immune pressure. Across modalities, the available evidence demonstrates that combination therapy is both biologically rational and clinically promising, yet major questions remain regarding patient selection, treatment sequencing, comparative efficacy, and appropriate clinical endpoints [12,38].

### 5.1. Synthesis Across Locoregional Modalities

Ablation combined with systemic therapy has produced some of the most consistent early signals of enhanced efficacy. Nearly all completed studies pairing ablation with agents such as sorafenib, lenvatinib, and nivolumab have reported improvements in PFS or OS compared to monotherapy (Table 3) [98,99,100,102,103,104,105,106,107,108]. However, sample sizes, timing of systemic therapy, and inclusion criteria varied widely. The recurrence patterns seen after ablation monotherapy demonstrate the need for systemic reinforcement of treatment durability. The trials reviewed collectively support the concept that ablation serves as an immune-priming event that systemic therapy can sustain or amplify, though heterogeneity in trial design limits cross-study comparisons. NIVOLVE, Lyu et al. (NCT03939975, Phase II, China), and related studies illustrate how trials using mechanistically similar agents can arrive at divergent estimates of PFS because of differences in cohort composition, timing of therapy, and definitions of recurrence [104,105].

TACE combination studies demonstrate a more mixed early history but a more promising contemporary trajectory. Phase II and III trials such as TACTICS, LAUNCH, IMMUTACE, EMERALD-1, and several single-arm efforts consistently show improved survival, prolonged time to progression, or meaningful response rates when TACE is paired with targeted agents or immunotherapies (Table 5) [116,121,122,123,126]. Trials that initially yielded equivocal results, such as SPACE and TACE 2, nonetheless established methodological precedents that shaped later trial design, including more precise definitions of progression and standardized imaging criteria [116,118,119,120,121,136]. The growing emphasis on pairing TACE with multi-agent systemic combinations reflects an evolution toward more sophisticated immunomodulatory strategies. Regimens such as nivolumab with ipilimumab or durvalumab with bevacizumab acknowledge the complex microenvironment of HCC and may ultimately expand treatment options for patients with intermediate or borderline advanced disease, particularly those with multifocal tumors or mildly impaired hepatic reserve.

Combination strategies involving TARE and systemic agents remain less extensively studied but are entering a period of accelerated growth. Early randomized studies pairing TARE with sorafenib, including SARAH, SIRveNIB, and SORAMIC, did not demonstrate meaningful improvements in OS or PFS (Table 7) [130,131,132]. These outcomes likely reflect broad or advanced inclusion criteria, variable exposure to prior TACE, and inconsistent definitions of progression. Nonetheless, these trials advanced the field by establishing that combinatorial therapy is tolerable and often associated with improved patient-reported quality of life. More recent single-arm and early-phase trials pairing TARE with PD-1 inhibitors have demonstrated survival exceeding 18 to 27 months in selected cohorts with manageable toxicity. The most promising upcoming trials employ multi-agent systemic combinations, optimized dosing schedules, and more granular stratification by tumor burden and liver function (Table 8). Trials such as ROWAN, EMERALD-Y90, and efforts evaluating tremelimumab and durvalumab suggest that radioembolization-specific immunogenic effects may be leveraged more effectively in the future.

### 5.2. Persistent Challenges in Evidence Interpretation

Despite substantial progress, several limitations complicate the interpretation of the current literature. The most prominent challenge is heterogeneity across trials. Studies differ not only in inclusion criteria, which range from very early to advanced disease, and from Child-Pugh A to B, but also in the timing of systemic therapy relative to LRT, the number of treatment cycles, and the duration of follow-up. Many trials use single-arm designs without monotherapy comparators, limiting conclusions about relative benefit. Endpoints also vary widely. Some studies report survival in months, others report survival as percentages, and others use definitions of progression that are not harmonized across institutions.

Another limitation stems from the inherent complexity of HCC biology. Tumor burden, vascular invasion, multifocality, and underlying liver dysfunction all influence treatment response but are inconsistently reported or controlled for across studies. These variables may help explain inconsistent results among trials using similar systemic agents. Differences in local procedural technique, including bead size in DEB-TACE, radiation dose in TARE, and ablative margin in RFA or MWA, introduce additional heterogeneity that complicates cross-study interpretation.

Patient selection and generalizability remain critical issues. Many early trials excluded patients with portal vein invasion or Child-Pugh B disease, although these populations are common in real-world practice. More recent trials deliberately include higher-risk patients, which may broaden the generalizability of future findings. Interpretation and generalizability of evidence is also inevitably influenced by global patterns in care. For example, while there is significantly more research evaluating TACE, these trials may be of lesser interests in areas where TARE is the predominant mode of embolization, such as North America.

### 5.3. Emerging Areas of Convergence

When examined collectively, the evidence suggests several important thematic patterns. Locoregional therapy is not simply cytoreductive; it dynamically reshapes immune and vascular signaling. Antiangiogenic therapies and PD-1 inhibitors appear particularly well suited for pairing with any of the available LRTs because they modulate the biologic responses induced by ischemia, radiation, or thermal injury. Timing also appears to influence outcomes, though no consistent pattern has emerged. Some studies suggest benefit from neoadjuvant systemic therapy, others from peri-procedural or adjuvant administration, and the heterogeneity of trial designs makes direct comparison difficult. What remains clear is that the question of sequencing cannot be resolved by the current evidence base and would benefit from further investigation.

Improved outcomes in downstaging, quality of life, and treatment tolerability highlight how combination strategies may not only extend survival but also expand curative options for select patients [26,38]. TACE and TARE combinations in particular have demonstrated the ability to downstage patients to resection or transplant. Standardizing the assessment and reporting of downstaging, quality of life, and functional endpoints will be essential for evaluating the full clinical value of combination strategies in future trials.

## 6. Future Directions

The rapid expansion of combination strategies in HCC has opened several avenues for clinical and scientific advancement. As locoregional and systemic therapies continue to evolve, future studies must address persistent uncertainties related to timing, sequencing, patient selection, and mechanistic predictors of response. The goal will be to move beyond proof of concept and define reproducible, evidence-based frameworks for integrating combination therapy across disease stages.

### 6.1. Optimizing Trial Design and Endpoint Selection

The next generation of trials will require more rigorous and uniform methodology. Standardized definitions of progression, objective response, downstaging, and recurrence are essential for meaningful comparison across studies. Trials should also incorporate pre-specified stratification by factors that consistently influence outcomes, including tumor burden, vascular invasion, and degree of hepatic dysfunction. Incorporating harmonized quality of life metrics may clarify whether certain combinations offer clinically meaningful benefit even when survival outcomes are similar.

Because many early and mid-stage studies relied on single-arm designs, there is a need for randomized trials that directly compare combination therapy to both monotherapy and other combination strategies. Trials such as EMERALD-Y90 (NCT06040099) and LEAP-012 (NCT04246177), which utilize multi-agent systemic regimens and more sophisticated cohort definitions, reflect the direction in which the field is moving.

### 6.2. Defining Optimal Sequencing and Timing

Resolving the sequencing question of systemic therapy relative to locoregional intervention will require a new generation of trials specifically designed to compare neoadjuvant, peri-procedural, and adjuvant strategies in parallel rather than against monotherapy or placebo alone. Such trials should incorporate standardized definitions of treatment timing and standardized response criteria to permit meaningful cross-study comparison.

Advances in functional imaging and liquid biopsy may eventually provide real-time biomarkers that guide when systemic therapy should be initiated, withheld, or escalated. Quantitative imaging approaches that characterize macrovascular invasion, perfusion, and tumor heterogeneity non-invasively could facilitate individualized sequencing decisions, moving the field beyond fixed protocols toward adaptive treatment strategies tailored to each patient’s evolving disease biology.

### 6.3. Expanding Curative Pathways and Downstaging

One of the most promising implications of combination therapy is the potential to expand eligibility for resection or transplantation. As more patients achieve radiographic downstaging or stabilization of liver function, understanding which regimens most reliably achieve downstaging becomes increasingly important. Trials with explicit downstaging endpoints, including ROWAN (NCT05063565), EMERALD-Y90, and other ongoing TACE-based studies, will be essential in defining how combination therapy may widen the window for curative intervention.

### 6.4. Integrating Immunologic and Genomic Biomarkers

As discussed, LRTs expose tumor-associated antigens and trigger immune system anti-neoplastic activity. Systemic therapies amplify or stabilize these shifts, yet biomarker development has lagged behind therapeutic innovation. Identifying genomic, proteomic, and immunologic predictors of response will be critical. Biomarkers related to WNT and beta-catenin activation, T-cell exhaustion signatures, and cytokine patterns may ultimately guide selection of systemic agents and predict which patients derive the greatest benefit from combined treatment [104].

### 6.5. Broadening Inclusion Criteria and Improving Generalizability

Real-world HCC frequently involves portal vein invasion, Child-Pugh B cirrhosis, and multifocal tumor burden. Historically, many trials excluded these patients. Expanding inclusion criteria, while maintaining safety, will improve applicability to clinical practice. More trials designed specifically for Child-Pugh B patients or those with segmental portal vein thrombus are needed to clarify when combination therapy is feasible and beneficial outside of idealized trial populations.

### 6.6. Leveraging Multi-Agent Combinations

Early trials combining two immune-based agents or pairing immunotherapy with antiangiogenic drugs suggest increasing interest in multi-agent systems. Future work must clarify whether dual or triple systemic combinations meaningfully outperform single-agent partners, and whether the added complexity is justified by improved survival, downstaging rates, or quality of life. Rational combinations grounded in tumor microenvironment biology, rather than empirical pairing, will likely yield the greatest benefit.

## 7. Conclusions

Combination strategies incorporating systemic therapies and LRTs represent a significant evolution in the management of HCC. The growing body of mechanistic, translational, and clinical evidence demonstrates that neither modality alone is sufficient to address the multifocality, microvascular invasion, and immune complexity characteristic of the disease. Locoregional therapies contribute rapid cytoreduction and immunogenic tumor injury, while systemic agents counteract escape pathways, suppress angiogenic rebound, and sustain antitumor immune activity. Together, these modalities offer a more comprehensive therapeutic framework capable of addressing tumor biology at multiple levels.

Completed studies across ablation and embolization methodologies illustrate the feasibility and safety of combination treatment, with several demonstrating improvements in PFS, OS, or both. Ongoing trials are refining questions related to timing, treatment sequence, biomarker selection, and multi-agent systemic regimens. These efforts reflect a broad recognition that optimized integration of therapies may expand access to curative options, improve downstaging success, and deliver more durable disease control.

As the field advances, the most impactful progress will arise from rigorous trial design, standardized endpoints, effective therapeutic combinations, and a commitment to addressing the needs of patients with more advanced liver dysfunction. Continued refinement of these strategies will be essential for translating the promise of combination therapy into routine clinical practice and improving long-term outcomes for patients with HCC.

## Figures and Tables

**Table 1:** Summary of BCLC and Child-Pugh Classification system.

BCLC Staging			
Description	Stage	Criteria	Treatments Indicated
Very early stage	0	Single tumor (≤2 cm), preserved liver function, patient is active with no performance restriction	Ablation, Resection
Early stage	A	Single tumor OR ≤3 tumors (≤3 cm, each), preserved liver function, patient is active with no performance restriction	Ablation, Resection, or Transplant
Intermediate stage	B	Multiple tumors, preserved liver function, patient is active with no performance restriction	Transplant, TACE/TARE, Systemic therapies
Advanced stage	C	Metastasis ± invasion of portal vasculature, preserved liver function, patient has mild impact to daily activities or is capable of self-care, but not work activities	Systemic Therapies
Terminal stage	D	Any tumor burden, end stage liver function, patient can perform limited self-care, or is completely disabled	Palliative Care
Child-Pugh Classification			
Classification Criteria	Class	Points	Severity
	A	5–6	Least
	B	7–9	Moderate
	C	10–15	Most
Points Criteria	1 point	2 points	3 points
Albumin (g/dL)	>3.5	2.8–3.5	<2.8
Ascites	None	Mild to moderate (responds to diuretics)	Severe (unresponsive to diuretics)
Bilirubin (mg/dL)	<2	2–3	>3
Encephalopathy	None	Mild to moderate	Severe
Prothrombin time increase (seconds) OR International normalized ratio	<4	4–6	>6
	<1.7	1.7–2.3	>2.3

**Table 2:** Summary of immunotherapies and LRT modalities.

Systemic Chemotherapies				
**Treatment Modality**		**Mechanism of Action**	**Comparative Efficacy**	**Common Side Effects**
Multikinase Inhibitors	Sorafenib, Lenvatinib, Regorafenib, Cabozantinib, Sunitinib	Inhibits serine-threonine kinases and VEGF receptors [9,10]	Sorafenib has shown increased OS in SHARP, GIDEON, and INSIGHT trials [10,12]	Hand-foot syndrome, fatigue, GI issues (nausea, vomiting, diarrhea) [9,10,12]
Immune Checkpoint Inhibitors	Pembrolizumab, Nivolumab, Toripalimab, Camrelizumab, Sintilimab, Tislelizumab	Enhances T-cell activity by blocking PD-1 [15]	Comparable OS to sorafenib but with longer PFS and time-to-progression [16,17,18,19]	Hypertension, hypothyroidism, proteinuria [16]
	Atezolizumab, Durvalumab	Enhances T-cell activity by blocking PD-L1 [15]		
	Ipilimumab, Tremelimumab	Activates cytotoxic T-cells by inhibiting CTLA-4 [17,18]	Combination with nivolumab shown effective; however, immune-related adverse effects noted [17,18,19,20]	Immune-mediated hepatitis, colitis, dermatitis [20,21,22,23]
Angiogenesis Inhibitors	Bevacizumab, Ramucirumab	Inhibits VEGF and tumor angiogenesis [24]	Increased OS post-sorafenib in KEYNOTE-240 trial [16]. Combination with atezolizumab increases OS compared to sorafenib [24]	Rash, immune-related adverse effects, GI issues (nausea, vomiting, diarrhea) [24,25]
**Ablative Techniques**				
Treatment Modality	Mechanism of Action [26]	Indications [26]	Comparative Efficacy	Common Side Effects [26,27,28,29]
Radiofrequency Ablation (RFA)	Heat-induced coagulative necrosis via electrical current	Very early and early-stage HCC (BCLC 0 and A)	Comparable OS to surgical resection for tumors <5 cm; shorter procedure times than cryoablation [30,31,32,33]	Abdominal pain, bleeding, thrombosis, fever, heat sink effect near large vessels
Cryoablation	Freezing and thawing cycles causing cellular damage	Very early and early-stage HCC (BCLC 0 and A), Tumors near biliary/GI structures, small tumors	Less effective for larger tumors (>3–5 cm); better suited for tumors near critical structures [26]	Cryoshock (rare), tumor lysis syndrome, fever, pain, biliary injury
Microwave Ablation (MWA)	Microwave-induced thermal injury	Very early and early-stage HCC (BCLC 0 and A) Multiple small lesions, especially in vascular areas	Similar OS to RFA but with fewer insertions and clearer ablation boundaries [26]	Vascular complications (bleeding, biliary injury), fever, pain, mortality rate around 0.36%
**Transarterial Embolization (TAE)**				
Treatment Modality	Mechanism of Action	Indications	Comparative Efficacy	Common Side Effects
TAE	Embolic agents induce tumor ischemia [34]	Early and intermediate-stage HCC (BCLC A, B) [34]	Median OS: 1–3 years, depending on liver function and tumor burden [35]	PES (fever, pain, nausea), bile duct injury, pulmonary embolism [36,37,38]
TACE	Chemotherapy combined with embolization [39]	Early and intermediate-stage HCC (BCLC A, B) [39]	Higher response rates compared to TAE; prolonged time to progression [40,41,42,43]	Increased risk of hepatic toxicities, severe PES, increased infection rates [44]
TARE	Yttrium-90 microspheres deliver targeted radiation [45]	Early and intermediate-stage HCC (BCLC A, B) [46]	Similar OS to TACE but with fewer toxic effects and longer time to progression [47,48,49,50,51]	Radioembolization-induced liver disease, radiation pneumonitis [52,53]

**Table 3:** Summary of completed RFA/immunotherapy studies. (RFSR: recurrence-free survival rate; RFS: recurrence free survival).

Trial	Status	Treatment	Study Size	Population Characteristics	OS (or RFSR)	PFS or (RFS)	Most Common Adverse Effects	Combination Therapy Superior to Monotherapy?	Additional Details
Gong et al. [98]	Completed	Sorafenib + RFA	90	BCLC 0-A	35%	12.3 months	Bleeding, diarrhea ascites, hand-foot reaction	Yes	n/a
Fukuda et al. [99]			152	BCLC 0-A	n/a	17.0 months		Yes	Ablation areas were smaller in patients who had also received sorafenib
Feng et al. [100]			128	BCLC 0-B	1y: 85.6%; 3y: 58.7%	n/a		Yes	Tumor recurrence was lower in combinatorial groups
Kan et al. [102]			62	Tumor between 3.1–5.0 cm; BCLC B or C; Child-Pugh A or B; no previous treatment	n/a	17.0 months		Yes	Tumor recurrence was lower in combinatorial groups
Wang et al. [103]	Completed	Lenvatinib + RFA	22	BCLC B; Child-Pugh A	1y: 100%; 2y: 80%	12.5 months; 1y: 74.1%	Appetite loss, elevated liver enzymes	Yes	n/a
NIVOLVE [104]	Completed (Phase II)	PD-1 inhibition + RFA	55	Child-Pugh A	1y: 78.6%	26.3 months	Fatigue, abdominal pain, nausea or vomiting, rash, elevated liver enzymes, elevated leukocytes	Single-arm design	n/a
Lyu et al. [105]	Completed (Phase II)		50	Child-Pugh A; unacceptable toxicity to sorafenib	16.9 months	5 months		Single-arm design	6.1 months TTP
Zhou et al. [106]	Completed (Phase I/II)		146	Child-Pugh A	18.4 months	7.1 months		Yes	Initial stage of the study demonstrated that doses of immunotherapy 3 days after ablation had a higher response rate than 14 days after. The data compared combinatorial therapy from 3-day group to systemic monotherapy.
Wang et al. [107]	Completed		127	BCLC 0-A	1y: 92.7%	9.0 months		Yes	n/a
Wen et al. [108]	Completed		40	BCLC 0-A; Child-Pugh A; recurrent HCC with prior RFA	1y: 62.7% (RFSR)	15.4 months (RFS)		Yes	n/a
Duffy et al. [109]	Completed	CTLA-4 inhibition + RFA	32	BCLC C; Child-Pugh A or B	12.3 months	6m: 57.1%; 1y: 33.1%	Pruritic rash, increased liver enzymes, hyperbilirubinemia	n/a	7.4 months TTP; Established that TACE and ablation performed similarly

**Table 4:** Summary of ongoing RFA/immunotherapy studies.

Trial	Phase	Estimated Completion	Treatment	Study Size	Population Characteristics	Study Arm(s)	Primary Endpoint	Secondary Endpoint
NIVOLEP (NCT03630640)	II	2023 (results not yet available)	Nivolumab + ablation	43	BCLC A; Child-Pugh A	IV nivolumab up to 1 year after LRT	RFS	OS, safety
NCT04652440	I/II	2024 (results not yet available)	Tislelizumab + ablation	30	BCLC A or B; Child-Pugh A	SQ tislelizumab 1 day before LRT then every 3 weeks for 3 cycles	Safety	ORR, OS, PFS
NCT04663035	II	2025	Tislelizumab + ablation	120	BCLC 0 or A; Child-Pugh A	SQ tislelizumab within 1 week of LRT for up to 1 year v. LRT monotherapy	RFS	OS, TTR, safety
NCT04150744	II	2026	Carrizumab + ablation	120	BCLC B or C; Child-Pugh A or B; no prior systemic therapy	RFA + carrizumab v. carrizumab alone	PFS	ORR, OS
Checkmate 9DX (NCT03383458)	III	2025	Nivolumab + ablation	545	Child-Pugh A	Nivolumab after RFA v. placebo after RFA	RFS	OS, TTR
KEYNOTE-937 (NCT03867084)	III	2029	Pembrolizumab + ablation	950	Child-Pugh A	Pembrolizumab after RFA v. placebo after RFA	RFS, OS	Safety, Quality of life questionnaire
IMbrave050 (NCT04102098)	III	2027	(Atezolizumab or Bevacizumab) + ablation	668	Child-Pugh A; ablation 4–12 weeks before randomization	Atezolizumab + bevacizumab v. placebo	RFS	OS, TTR

**Table 6:** Summary of ongoing TACE/immunotherapy trials (ORR: overall response rate).

Trial	Phase	Estimated Completion	Treatment	Study Size	Population Characteristics	Study Arm(s)	Primary Endpoint	Secondary Endpoint
NCT04297280	II	2025	TACE + Sintilimab	25	BCLC B-C; Child-Pugh A	Sintilimab administered 14 days after TACE until disease progression noted	ORR	PFS, OS, safety
NCT03143270	II	2026	DEB-TACE + Nivolumab	20	BCLC B; Child-Pugh A; unresectable HCC	1: Nivolumab administered 14 days after TACE, then every 2 weeks for up to 1 year 2: Nivolumab administered 4 weeks before TACE, then every 2 weeks for up to 1 year 3: Nivolumab administered 4 weeks before TACE, including the day of, then every 2 weeks for up to 1 year	Safety	n/a
TACE-3 (NCT04268888)	II/III	2026	TACE ± Nivolumab	522	Child-Pugh A; unresectable HCC	IV nivolumab + TACE vs. TACE/TAE	OS, TTP	Response, PFS, quality of life, safety
EMERALD-1 (NCT03778957)	III	2026	TACE ± (Durvalumab ± Bevacizumab)	724	Child-Pugh A-B7; not amenable to surgery but amenable to TACE	TACE alone vs. TACE + Durvalumab vs. TACE + Durvalumab + Bevacizumab	PFS	OS, quality of life, symptoms
EMERALD-2 (NCT03847428)	III	2027	Resection/LRT ± (Durvalumab + Bevacizumab)	908	Child-Pugh 5–6; high risk of recurrence post-treatment, including surgical resection/LRT	IV durvalumab + bevacizumab, IV durvalumab, or placebo	RFS	OS, TTR
EMERALD-3 (NCT05301842)	III	2027	TACE ± (Durvalumab + Tremelimumab ± Lenvatinib)	760	Child-Pugh A; not amenable to surgical resection but amenable to TACE	IV durvalumab + bevacizumab, IV durvalumab, or placebo	RFS	OS, TTR
TALENTACE (NCT04712643)	III	2029	TACE ± (Atezolizumab + Bevacizumab)	342	Child-Pugh A; no prior treatment	IV atezolizumab every 3 weeks + bevacizumab every 3 weeks + TACE vs. TACE	PFS, OS	TTP, ORR, metastasis, safety
LEAP-012 (NCT04246177)	III	2029	TACE ± (Lenvatinib + Pembrolizumab)	450	HCC not amenable to curative treatment	Oral lenvatinib for 3 weeks + IV pembrolizumab every 6 weeks + TACE vs. TACE	PFS, OS	TTP, ORR, metastasis, safety
NCT05608200	III	2026	(TACE + Lenvatinib) ± Sintilimab	427	BCLC B-C; Child-Pugh A or B; history of tumor recurrence after resection or ablation	Oral lenvatinib and IV sintilimab within 1 week of receiving TACE vs. oral lenvatinib within 1 week of receiving TACE	OS	PFS, TTP, ORR, safety
NCT04992143	II	2023 (results not yet available)	TACE + Tislelizumab + Sorafenib	20	BCLC C; Child-Pugh A; no previous systemic therapy	Tislelizumab and sorafenib within 1 week of receiving TACE	OS	PFS, TTP, ORR

**Table 7:** Summary of completed TARE/immunotherapy trials, including notable trials that directly compared TARE to systemic therapy.

Trial	Status	Treatment	Study Size	Population Characteristics	OS (or RFSR)	PFS (or RFS)	Most Common Adverse Effects	Did Combinatorial Treatment Outperform Monotherapy?	Additional Details
SARAH [130]	Completed (Phase III)	Sorafenib vs. TARE	467	BCLC C; Child-Pugh A; not amenable to curative procedure; previous history >1 unsuccessful TACE	9.9 months (sorafenib) vs. 8.0 months (TARE)	n/a	Fatigue, weight loss, nausea, vomiting, diarrhea	No significant differences between monotherapies	Quality of life was rated higher in TARE group
SIRveNIB [131]	Completed (Phase III)		360	BCLC B-C; Child-Pugh A or B; not amenable to curative procedure	10.0 months (sorafenib) vs. 8.8 months (TARE)	n/a	Ascites, abdominal pain, anemia, radiation hepatitis	No significant differences between monotherapies	Improved safety in TARE group
SORAMIC [132]	Completed (Phase II)	Sorafenib + TARE	424	BCLC A-C; Child-Pugh A or B; no previous systemic therapy; unresectable HCC	14.0 months	n/a	Hand foot syndrome, hyperbilirubinemia	No	Improved quality of life in TARE group
Fenton et al. [133]	Completed (Phase I)	Nivolumab + TARE	27	Child-Pugh A or B; no previous systemic therapy; unresectable HCC	n/a	n/a	Elevated liver enzymes	n/a	Established maximum tolerated dose of nivolumab (240 mg, 2 weeks after TARE), disease control rate (82%)
NASIR-HCC [134]	Completed (Phase II)		41	Child-Pugh A	20.9 months	n/a	n/a	Single Arm	8.8 months TTP, four patients were downstaged to receive a partial hepatectomy
Yu et al. [135]	Completed (Phase I)	Pembrolizumab + TARE	30	Child-Pugh A or B; no previous systemic therapy; unresectable HCC	27.3 months	9.9 months	Fatigue, elevated liver enzymes, elevated bilirubin, abdominal pain	Single Arm	Study specifically selected for poor prognosis patients

**Table 8:** Summary of ongoing TARE/immunotherapy trials.

Trial	Phase	Estimated Completion	Treatment	Study Size	Population Characteristics	Study Arm(s)	Primary Endpoint	Secondary Endpoint
NCT03033446	II	2025	Nivolumab + TARE	40	Child-Pugh A; unresectable HCC; no prior TARE	IV nivolumab 3 weeks after TARE, repeated every 2 weeks	ORR	TTP, PFS, OS, safety, quality of life
NCT04605731	II	2025	Tremelimumab + Durvalumab + TARE	32	BCLC B-C; Child-Pugh A; unresectable HCC	IV tremelimumab and durvalumab given 2 weeks before TARE, then durvalumab every 4 weeks for up to 1 year	ORR, Safety	PFS, OS
NCT04522544	II	2026	Tremelimumab + Durvalumab + TARE	55	Child-Pugh A; unresectable HCC	IV tremelimumab and durvalumab given alongside standard-of-care TARE or TACE, then durvalumab every 4 weeks for up to 1 year	ORR	PFS, OS, safety, quality of life
ROWAN (NCT05063565)	II	2027	Tremelimumab + Durvalumab + TARE	100	BCLC B-C; Child-Pugh A; unresectable HCC	IV tremelimumab and durvalumab given after TARE, then durvalumab every 4 weeks for up to 18 months	ORR	PFS, OS, safety, quality of life, change in liver function, successfully downstaged patients
EMERALD-Y90 (NCT06040099)	II	2026	Bevacizumab + Durvalumab + TARE	100	Child-Pugh A; unresectable HCC	IV durvalumab + bevacizumab with TARE	PFS	ORR, OS, safety

## Data Availability

No new data were created or analyzed in this study.

## References

[1] Rumgay H., Arnold M., Ferlay J., Lesi O., Cabasag C.J., Vignat J., Laversanne M., McGlynn K.A., Soerjomataram I. (2022). Global burden of primary liver cancer in 2020 and predictions to 2040. J. Hepatol..

[2] Wang J., Qiu K., Zhou S., Gan Y., Jiang K., Wang D., Wang H. (2025). Risk factors for hepatocellular carcinoma: An umbrella review of systematic review and meta-analysis. Ann. Med..

[3] Liu P.-H., Hsu C.-Y., Hsia C.-Y., Lee Y.-H., Su C.-W., Huang Y.-H., Lee F.-Y., Lin H.-C., Huo T.-I. (2016). Prognosis of hepatocellular carcinoma: Assessment of eleven staging systems. J. Hepatol..

[4] Kinoshita A., Onoda H., Fushiya N., Koike K., Nishino H., Tajiri H. (2015). Staging systems for hepatocellular carcinoma: Current status and future perspectives. World J. Hepatol..

[5] Bruix J., Reig M., Sherman M. (2016). Evidence-Based Diagnosis, Staging, and Treatment of Patients With Hepatocellular Carcinoma. Gastroenterology.

[6] Reig M., Forner A., Rimola J., Ferrer-Fàbrega J., Burrel M., Garcia-Criado Á., Kelley R.K., Galle P.R., Mazzaferro V., Salem R. (2022). BCLC strategy for prognosis prediction and treatment recommendation: The 2022 update. J. Hepatol..

[7] Kim D.-H. (2022). Combination of interventional oncology local therapies and immunotherapy for the treatment of hepatocellular carcinoma. J. Liver Cancer.

[8] Zhang W.-C., Du K.-Y., Yu S.-F., Guo X.-E., Yu H.-X., Wu D.-Y., Pan C., Zhang C., Wu J., Bian L.-F. (2025). Systemic chemotherapy improves outcome of hepatocellular carcinoma patients treated with transarterial chemoembolization. Hepatobiliary Pancreat. Dis. Int. HBPD INT.

[9] Fan Y., Xue H., Zheng H. (2022). Systemic Therapy for Hepatocellular Carcinoma: Current Updates and Outlook. J. Hepatocell. Carcinoma.

[10] Llovet J.M., Ricci S., Mazzaferro V., Hilgard P., Gane E., Blanc J.-F., de Oliveira A.C., Santoro A., Raoul J.-L., Forner A. (2008). Sorafenib in advanced hepatocellular carcinoma. N. Engl. J. Med..

[11] Gong L., Giacomini M.M., Giacomini C., Maitland M.L., Altman R.B., Klein T.E. (2017). PharmGKB summary: Sorafenib pathways. Pharmacogenet. Genom..

[12] Bajestani N., Wu G., Hussein A., Makary M.S. (2024). Examining the Efficacy and Safety of Combined Locoregional Therapy and Immunotherapy in Treating Hepatocellular Carcinoma. Biomedicines.

[13] Ganten T.M., Stauber R.E., Schott E., Malfertheiner P., Buder R., Galle P.R., Göhler T., Walther M., Koschny R., Gerken G. (2017). Sorafenib in Patients with Hepatocellular Carcinoma-Results of the Observational INSIGHT Study. Clin. Cancer Res. Off. J. Am. Assoc. Cancer Res..

[14] Singal A.G., Llovet J.M., Yarchoan M., Mehta N., Heimbach J.K., Dawson L.A., Jou J.H., Kulik L.M., Agopian V.G., Marrero J.A. (2023). AASLD Practice Guidance on prevention, diagnosis, and treatment of hepatocellular carcinoma. Hepatology.

[15] Wang K., Wang C., Jiang H., Zhang Y., Lin W., Mo J., Jin C. (2021). Combination of Ablation and Immunotherapy for Hepatocellular Carcinoma: Where We Are and Where to Go. Front. Immunol..

[16] Finn R.S., Ryoo B.-Y., Merle P., Kudo M., Bouattour M., Lim H.Y., Breder V., Edeline J., Chao Y., Ogasawara S. (2020). Pembrolizumab As Second-Line Therapy in Patients With Advanced Hepatocellular Carcinoma in KEYNOTE-240: A Randomized, Double-Blind, Phase III Trial. J. Clin. Oncol. Off. J. Am. Soc. Clin. Oncol..

[17] Yau T., Kang Y.-K., Kim T.-Y., El-Khoueiry A.B., Santoro A., Sangro B., Melero I., Kudo M., Hou M.-M., Matilla A. (2020). Efficacy and Safety of Nivolumab Plus Ipilimumab in Patients With Advanced Hepatocellular Carcinoma Previously Treated With Sorafenib: The CheckMate 040 Randomized Clinical Trial. JAMA Oncol..

[18] Tsang J., Wong J.S.L., Kwok G.G.W., Li B., Cho W., Leung R., Chiu J., Cheung T., Yau T. (2021). Nivolumab + Ipilimumab for patients with hepatocellular carcinoma previously treated with Sorafenib. Expert Rev. Gastroenterol. Hepatol..

[19] Yau T., Park J.-W., Finn R.S., Cheng A.-L., Mathurin P., Edeline J., Kudo M., Harding J.J., Merle P., Rosmorduc O. (2022). Nivolumab versus sorafenib in advanced hepatocellular carcinoma (CheckMate 459): A randomised, multicentre, open-label, phase 3 trial. Lancet. Oncol..

[20] Yau T., Galle P.R., Decaens T., Sangro B., Qin S., da Fonseca L.G., Karachiwala H., Blanc J.-F., Park J.-W., Gane E. (2025). Nivolumab plus ipilimumab versus lenvatinib or sorafenib as first-line treatment for unresectable hepatocellular carcinoma (CheckMate 9DW): An open-label, randomised, phase 3 trial. Lancet.

[21] Abou-Alfa G.K., Chan S.L., Kudo M., Lau G., Kelley R.K., Furuse J., Sukeepaisarnjaroen W., Kang Y.-K., Dao T.V., De Toni E.N. (2022). Phase 3 randomized, open-label, multicenter study of tremelimumab (T) and durvalumab (D) as first-line therapy in patients (pts) with unresectable hepatocellular carcinoma (uHCC): HIMALAYA. J. Clin. Oncol..

[22] Sangro B., Chan S.L., Kelley R.K., Lau G., Kudo M., Sukeepaisarnjaroen W., Yarchoan M., De Toni E.N., Furuse J., Kang Y.K. (2024). Four-year overall survival update from the phase III HIMALAYA study of tremelimumab plus durvalumab in unresectable hepatocellular carcinoma. Ann. Oncol. Off. J. Eur. Soc. Med. Oncol..

[23] Rimassa L., Chan S.L., Sangro B., Lau G., Kudo M., Reig M., Breder V., Ryu M.-H., Ostapenko Y., Sukeepaisarnjaroen W. (2025). Five-year overall survival update from the HIMALAYA study of tremelimumab plus durvalumab in unresectable HCC. J. Hepatol..

[24] Cheng A.-L., Qin S., Ikeda M., Galle P.R., Ducreux M., Kim T.-Y., Lim H.Y., Kudo M., Breder V., Merle P. (2022). Updated efficacy and safety data from IMbrave150: Atezolizumab plus bevacizumab vs. sorafenib for unresectable hepatocellular carcinoma. J. Hepatol..

[25] Zhu A.X., Finn R.S., Kang Y.-K., Yen C.-J., Galle P.R., Llovet J.M., Assenat E., Brandi G., Motomura K., Ohno I. (2021). Serum alpha-fetoprotein and clinical outcomes in patients with advanced hepatocellular carcinoma treated with ramucirumab. Br. J. Cancer.

[26] Fazlollahi F., Makary M.S. (2025). Precision oncology: The role of minimally-invasive ablation therapy in the management of solid organ tumors. World J. Radiol..

[27] Kuroda H., Nagasawa T., Fujiwara Y., Sato H., Abe T., Kooka Y., Endo K., Oikawa T., Sawara K., Takikawa Y. (2021). Comparing the Safety and Efficacy of Microwave Ablation Using ThermosphereTM Technology versus Radiofrequency Ablation for Hepatocellular Carcinoma: A Propensity Score-Matched Analysis. Cancers.

[28] Ko S.E., Lee M.W., Rhim H., Kang T.W., Song K.D., Cha D.I., Lim H.K. (2020). Comparison of procedure-related complications between percutaneous cryoablation and radiofrequency ablation for treating periductal hepatocellular carcinoma. Int. J. Hyperth. Off. J. Eur. Soc. Hyperthermic Oncol. N. Am. Hyperth. Group.

[29] Chen L., Ren Y., Sun T., Cao Y., Yan L., Zhang W., Ouyang T., Zheng C. (2021). The efficacy of radiofrequency ablation versus cryoablation in the treatment of single hepatocellular carcinoma: A population-based study. Cancer Med..

[30] Wang Y., Luo Q., Li Y., Deng S., Wei S., Li X. (2014). Radiofrequency ablation versus hepatic resection for small hepatocellular carcinomas: A meta-analysis of randomized and nonrandomized controlled trials. PLoS ONE.

[31] Yang W., Li X., Tan X. (2025). Microwave ablation versus radiofrequency ablation for hepatocellular carcinoma: A propensity score matching and inverse probability weighting analysis. Int. J. Hyperth. Off. J. Eur. Soc. Hyperthermic Oncol. N. Am. Hyperth. Group.

[32] Bruix J., Gores G.J., Mazzaferro V. (2014). Hepatocellular carcinoma: Clinical frontiers and perspectives. Gut.

[33] Han X., Ni J.-Y., Li S.-L., Deng H.-X., Liang H.-M., Xu Y.-Y., Huang Z.-M., Zhang T.-Q., Huang J.-H. (2021). Radiofrequency versus microwave ablation for hepatocellular carcinoma within the Milan criteria in challenging locations: A retrospective controlled study. Abdom. Radiol..

[34] Boily G., Villeneuve J.-P., Lacoursière L., Chaudhury P., Couture F., Ouellet J.-F., Lapointe R., Goulet S., Gervais N. (2015). Comité de l’évolution des pratiques en oncologie Transarterial embolization therapies for the treatment of hepatocellular carcinoma: CEPO review and clinical recommendations. HPB Off. J. Int. Hepato Pancreato Biliary Assoc..

[35] Lawson A., Kamarajah S.K., Parente A., Pufal K., Sundareyan R., Pawlik T.M., Ma Y.T., Shah T., Kharkhanis S., Dasari B.V.M. (2023). Outcomes of Transarterial Embolisation (TAE) vs. Transarterial Chemoembolisation (TACE) for Hepatocellular Carcinoma: A Systematic Review and Meta-Analysis. Cancers.

[36] Mason M.C., Massarweh N.N., Salami A., Sultenfuss M.A., Anaya D.A. (2015). Post-embolization syndrome as an early predictor of overall survival after transarterial chemoembolization for hepatocellular carcinoma. HPB Off. J. Int. Hepato Pancreato Biliary Assoc..

[37] Dhand S., Gupta R. (2011). Hepatic transcatheter arterial chemoembolization complicated by postembolization syndrome. Semin. Interv. Radiol..

[38] Jipa A.M., Makary M.S. (2024). Locoregional Therapies for Hepatobiliary Tumors: Contemporary Strategies and Novel Applications. Cancers.

[39] Lanza C., Ascenti V., Amato G.V., Pellegrino G., Triggiani S., Tintori J., Intrieri C., Angileri S.A., Biondetti P., Carriero S. (2025). All You Need to Know About TACE: A Comprehensive Review of Indications, Techniques, Efficacy, Limits, and Technical Advancement. J. Clin. Med..

[40] Malagari K., Pomoni M., Moschouris H., Bouma E., Koskinas J., Stefaniotou A., Marinis A., Kelekis A., Alexopoulou E., Chatziioannou A. (2012). Chemoembolization with doxorubicin-eluting beads for unresectable hepatocellular carcinoma: Five-year survival analysis. Cardiovasc. Interv. Radiol..

[41] Zane K.E., Nagib P.B., Jalil S., Mumtaz K., Makary M.S. (2022). Emerging curative-intent minimally-invasive therapies for hepatocellular carcinoma. World J. Hepatol..

[42] Lee M., Shin H.P. (2023). Efficacy of Transarterial Chemoembolization (TACE) for Early-Stage Hepatocellular Carcinoma. Med. (Kaunas Lith.).

[43] Bargellini I., Sacco R., Bozzi E., Bertini M., Ginanni B., Romano A., Cicorelli A., Tumino E., Federici G., Cioni R. (2012). Transarterial chemoembolization in very early and early-stage hepatocellular carcinoma patients excluded from curative treatment: A prospective cohort study. Eur. J. Radiol..

[44] Schindler P., Kaldewey D., Rennebaum F., Trebicka J., Pascher A., Wildgruber M., Köhler M., Masthoff M. (2024). Safety, efficacy, and survival of different transarterial chemoembolization techniques in the management of unresectable hepatocellular carcinoma: A comparative single-center analysis. J. Cancer Res. Clin. Oncol..

[45] Kallini J.R., Gabr A., Salem R., Lewandowski R.J. (2016). Transarterial Radioembolization with Yttrium-90 for the Treatment of Hepatocellular Carcinoma. Adv. Ther..

[46] Lau W.-Y., Kennedy A.S., Kim Y.H., Lai H.K., Lee R.-C., Leung T.W.T., Liu C.-S., Salem R., Sangro B., Shuter B. (2012). Patient selection and activity planning guide for selective internal radiotherapy with yttrium-90 resin microspheres. Int. J. Radiat. Oncol. Biol. Phys..

[47] Makary M.S., Bozer J., Miller E.D., Diaz D.A., Rikabi A. (2024). Long-term Clinical Outcomes of Yttrium-90 Transarterial Radioembolization for Hepatocellular Carcinoma: A 5-Year Institutional Experience. Acad. Radiol..

[48] Sangro B., Carpanese L., Cianni R., Golfieri R., Gasparini D., Ezziddin S., Paprottka P.M., Fiore F., Van Buskirk M., Bilbao J.I. (2011). European Network on Radioembolization with Yttrium-90 Resin Microspheres (ENRY). Survival after Yttrium-90 Resin Microsphere Radioembolization of Hepatocellular Carcinoma across Barcelona Clinic Liver Cancer Stages: A European Evaluation. Hepatol. Baltim. Md.

[49] Salem R., Johnson G.E., Kim E., Riaz A., Bishay V., Boucher E., Fowers K., Lewandowski R., Padia S.A. (2021). Yttrium-90 Radioembolization for the Treatment of Solitary, Unresectable HCC: The LEGACY Study. Hepatol. Baltim. Md.

[50] Hu T., Xu Q., Jia G., Wang T., Zuo C. (2025). A Bibliometric Analysis of 30 Years of Research on Transarterial Radioembolization (TARE) for Hepatocellular Carcinoma. Front. Pharmacol..

[51] Salem R., Lewandowski R.J., Kulik L., Wang E., Riaz A., Ryu R.K., Sato K.T., Gupta R., Nikolaidis P., Miller F.H. (2011). Radioembolization Results in Longer Time-to-Progression and Reduced Toxicity Compared with Chemoembolization in Patients with Hepatocellular Carcinoma. Gastroenterology.

[52] Kis B., Gyano M. (2025). Radiation Pneumonitis after Yttrium-90 Radioembolization: A Systematic Review. J. Vasc. Interv. Radiol..

[53] Wright C.L., Werner J.D., Tran J.M., Gates V.L., Rikabi A.A., Shah M.H., Salem R. (2012). Radiation Pneumonitis Following Yttrium-90 Radioembolization: Case Report and Literature Review. J. Vasc. Interv. Radiol. JVIR.

[54] Finn R.S., Kudo M., Cheng A.-L., Wyrwicz L., Ngan R.K.C., Blanc J.-F., Baron A.D., Vogel A., Ikeda M., Piscaglia F. (2021). Pharmacodynamic Biomarkers Predictive of Survival Benefit with Lenvatinib in Unresectable Hepatocellular Carcinoma: From the Phase III REFLECT Study. Clin. Cancer Res. Off. J. Am. Assoc. Cancer Res..

[55] Bruix J., Qin S., Merle P., Granito A., Huang Y.-H., Bodoky G., Pracht M., Yokosuka O., Rosmorduc O., Breder V. (2017). Regorafenib for patients with hepatocellular carcinoma who progressed on sorafenib treatment (RESORCE): A randomised, double-blind, placebo-controlled, phase 3 trial. Lancet.

[56] Abou-Alfa G.K., Meyer T., Cheng A.-L., El-Khoueiry A.B., Rimassa L., Ryoo B.-Y., Cicin I., Merle P., Chen Y., Park J.-W. (2018). Cabozantinib in Patients with Advanced and Progressing Hepatocellular Carcinoma. N. Engl. J. Med..

[57] El-Khoueiry A.B., Meyer T., Cheng A.-L., Rimassa L., Sen S., Milwee S., Kelley R.K., Abou-Alfa G.K. (2022). Safety and efficacy of cabozantinib for patients with advanced hepatocellular carcinoma who advanced to Child-Pugh B liver function at study week 8: A retrospective analysis of the CELESTIAL randomised controlled trial. BMC Cancer.

[58] Chau I., Peck-Radosavljevic M., Borg C., Malfertheiner P., Seitz J.F., Park J.O., Ryoo B.-Y., Yen C.-J., Kudo M., Poon R. (2017). Ramucirumab as second-line treatment in patients with advanced hepatocellular carcinoma following first-line therapy with sorafenib: Patient-focused outcome results from the randomised phase III REACH study. Eur. J. Cancer.

[59] Wu M.-C., Tang Z.-Y., Ye S.-L., Fan J., Qin S.-K., Yang J.-M., Chen M.-S., Chen M.-H., Lv M.-D., Ma K.-S. (2012). Expert consensus on local ablation therapies for primary liver cancer. Chin. Clin. Oncol..

[60] Granito A., Bolondi L. (2017). Non-transplant therapies for patients with hepatocellular carcinoma and Child-Pugh-Turcotte class B cirrhosis. Lancet. Oncol..

[61] Fang C., Cortis K., Yusuf G.T., Gregory S., Lewis D., Kane P., Peddu P. (2019). Complications from percutaneous microwave ablation of liver tumours: A pictorial review. Br. J. Radiol..

[62] Sun Q., Shi J., Ren C., Du Z., Shu G., Wang Y. (2020). Survival analysis following microwave ablation or surgical resection in patients with hepatocellular carcinoma conforming to the Milan criteria. Oncol. Lett..

[63] Yeo Y.-H., Kang Y.-N., Chen C., Lee T.-Y., Yeh C.-C., Huang T.-W., Wu C.-Y. (2024). Liver resection had better disease-free survival rates compared with radiofrequency ablation in hepatocellular carcinoma: A meta-analysis based on randomized clinical trials. Int. J. Surg..

[64] Ryu T., Takami Y., Wada Y., Saitsu H. (2022). Oncological Outcomes of Operative Microwave Ablation for Intermediate Stage Hepatocellular Carcinoma: Experience in 246 Consecutive Patients. J. Gastrointest. Surgery Off. J. Soc. Surg. Aliment. Tract.

[65] Eid R.A., Abdel Fattah A.M., Haseeb A.F., Hamed A.M., Shaker M.A. (2024). Percutaneous Radiofrequency Ablation for Stage B1 of Modified Bolondi’s Subclassification for Intermediate-Stage Hepatocellular Carcinoma. Egypt. Liver J..

[66] Katsanos K., Kitrou P., Spiliopoulos S., Maroulis I., Petsas T., Karnabatidis D. (2017). Comparative effectiveness of different transarterial embolization therapies alone or in combination with local ablative or adjuvant systemic treatments for unresectable hepatocellular carcinoma: A network meta-analysis of randomized controlled trials. PLoS ONE.

[67] Brown A.M., Kassab I., Massani M., Townsend W., Singal A.G., Soydal C., Moreno-Luna L., Roberts L.R., Chen V.L., Parikh N.D. (2023). TACE versus TARE for patients with hepatocellular carcinoma: Overall and individual patient level meta analysis. Cancer Med..

[68] Loffroy R., Desmyttere A.-S., Mouillot T., Pellegrinelli J., Facy O., Drouilllard A., Falvo N., Charles P.-E., Bardou M., Midulla M. (2021). Ten-year experience with arterial embolization for peptic ulcer bleeding: N-butyl cyanoacrylate glue versus other embolic agents. Eur. Radiol..

[69] Roth G.S., Benhamou M., Teyssier Y., Seigneurin A., Abousalihac M., Sengel C., Seror O., Ghelfi J., Ganne-Carrié N., Blaise L. (2021). Comparison of Trans-Arterial Chemoembolization and Bland Embolization for the Treatment of Hepatocellular Carcinoma: A Propensity Score Analysis. Cancers.

[70] Patel I.J., Rahim S., Davidson J.C., Hanks S.E., Tam A.L., Walker T.G., Wilkins L.R., Sarode R., Weinberg I. (2019). Society of Interventional Radiology Consensus Guidelines for the Periprocedural Management of Thrombotic and Bleeding Risk in Patients Undergoing Percutaneous Image-Guided Interventions-Part II: Recommendations: Endorsed by the Canadian Association for In. J. Vasc. Interv. Radiol. JVIR.

[71] Lanza E., Muglia R., Bolengo I., Poretti D., D’Antuono F., Ceriani R., Torzilli G., Pedicini V. (2020). Survival analysis of 230 patients with unresectable hepatocellular carcinoma treated with bland transarterial embolization. PLoS ONE.

[72] Makary M.S., Khandpur U., Cloyd J.M., Mumtaz K., Dowell J.D. (2020). Locoregional Therapy Approaches for Hepatocellular Carcinoma: Recent Advances and Management Strategies. Cancers.

[73] Miyayama S. (2020). Treatment Strategy of Transarterial Chemoembolization for Hepatocellular Carcinoma. Appl. Sci..

[74] de Baere T., Arai Y., Lencioni R., Geschwind J.-F., Rilling W., Salem R., Matsui O., Soulen M.C. (2016). Treatment of Liver Tumors with Lipiodol TACE: Technical Recommendations from Experts Opinion. Cardiovasc. Interv. Radiol..

[75] Hatanaka T., Arai H., Kakizaki S. (2018). Balloon-occluded transcatheter arterial chemoembolization for hepatocellular carcinoma. World J. Hepatol..

[76] Cho Y., Choi J.W., Kwon H., Kim K.Y., Lee B.C., Chu H.H., Lee D.H., Lee H.A., Kim G.M., Oh J.S. (2023). Transarterial chemoembolization for hepatocellular carcinoma: 2023 expert consensus-based practical recommendations of the Korean Liver Cancer Association. J. Liver Cancer.

[77] Badar W., Yu Q., Patel M., Ahmed O. (2024). Transarterial Radioembolization for Management of Hepatocellular Carcinoma. Oncologist.

[78] Keskin O., Soydal C., Deda X., Manti B., Tuzun A. (2015). Systemic Radiopharmaceutical Agents (Sm-153) may be Dangerous in Hepatacellular Carcinoma. World J. Nucl. Med..

[79] Datta Gupta S.S., Shamim S.A., Gamanagatti S., Gupta P., Khan M.A., Mallia M.B., Chirayil V., Dash A., Bal C. (2024). Re-188 lipiodol in hepatocellular carcinoma with portal vein thrombosis: A pilot study using novel chelating agent N-DEDC and its comparison with (A)HDD. Nucl. Med. Commun..

[80] Furtado R.V., Ha L., Clarke S., Sandroussi C. (2015). Adjuvant Iodine (131) Lipiodol after Resection of Hepatocellular Carcinoma. J. Oncol..

[81] Lewandowski R.J., Gabr A., Abouchaleh N., Ali R., Al Asadi A., Mora R.A., Kulik L., Ganger D., Desai K., Thornburg B. (2018). Radiation Segmentectomy: Potential Curative Therapy for Early Hepatocellular Carcinoma. Radiology.

[82] Smits M.L.J., Nijsen J.F.W., van den Bosch M.A.A.J., Lam M.G.E.H., Vente M.A.D., Huijbregts J.E., van het Schip A.D., Elschot M., Bult W., de Jong H.W.A.M. (2010). Holmium-166 radioembolization for the treatment of patients with liver metastases: Design of the phase I HEPAR trial. J. Exp. Clin. Cancer Res. CR.

[83] Stella M., Braat A.J.A.T., van Rooij R., de Jong H.W.A.M., Lam M.G.E.H. (2022). Holmium-166 Radioembolization: Current Status and Future Prospective. Cardiovasc. Interv. Radiol..

[84] Hendriks P., Rietbergen D.D.D., van Erkel A.R., Coenraad M.J., Arntz M.J., Bennink R.J., Braat A.E., Crobach S., van Delden O.M., Dibbets-Schneider P. (2024). Adjuvant holmium-166 radioembolization after radiofrequency ablation in early-stage hepatocellular carcinoma patients: A dose-finding study (HORA EST HCC trial). Eur. J. Nucl. Med. Mol. Imaging.

[85] Salem R., Thurston K.G. (2006). Radioembolization with yttrium-90 microspheres: A state-of-the-art brachytherapy treatment for primary and secondary liver malignancies: Part 3: Comprehensive literature review and future direction. J. Vasc. Interv. Radiol. JVIR.

[86] Lu W., Zhang T., Xia F., Huang X., Gao F. (2024). Transarterial radioembolization versus chemoembolization for hepatocellular carcinoma: A meta-analysis. Front. Oncol..

[87] Choi J.W., Kim H.-C. (2022). Radioembolization for hepatocellular carcinoma: What clinicians need to know. J. Liver Cancer.

[88] Lu J., Zhang X.-P., Zhong B.-Y., Lau W.Y., Madoff D.C., Davidson J.C., Qi X., Cheng S.-Q., Teng G.-J. (2019). Management of patients with hepatocellular carcinoma and portal vein tumour thrombosis: Comparing east and west. lancet. Gastroenterol. Hepatol..

[89] Singh P., Anil G. (2014). Yttrium-90 Radioembolization of Liver Tumors: What Do the Images Tell Us?. Cancer Imaging.

[90] Lim L., Gibbs P., Yip D., Shapiro J.D., Dowling R., Smith D., Little A., Bailey W., Liechtenstein M. (2005). Prospective Study of Treatment with Selective Internal Radiation Therapy Spheres in Patients with Unresectable Primary or Secondary Hepatic Malignancies. Intern. Med. J..

[91] Stella M., van Rooij R., Lam M.G.E.H., de Jong H.W.A.M., Braat A.J.A.T. (2022). Lung Dose Measured on Postradioembolization 90Y PET/CT and Incidence of Radiation Pneumonitis. J. Nucl. Med. Off. Publ. Soc. Nucl. Med..

[92] Kim D.J., Clark P.J., Heimbach J., Rosen C., Sanchez W., Watt K., Charlton M.R. (2014). Recurrence of Hepatocellular Carcinoma: Importance of mRECIST Response to Chemoembolization and Tumor Size. Am. J. Transpl..

[93] Briody H., Duong D., Yeoh S.W., Hodgson R., Yong T.L., Hannah A., Lee M.J., Leong S., Maingard J., Asadi H. (2023). Radioembolization for Treatment of Hepatocellular Carcinoma: Current Evidence and Patterns of Utilization. J. Vasc. Interv. Radiol..

[94] Rochigneux P., Nault J.-C., Mallet F., Chretien A.-S., Barget N., Garcia A.J., Del Pozo L., Bourcier V., Blaise L., Grando-Lemaire V. (2019). Dynamic of Systemic Immunity and Its Impact on Tumor Recurrence after Radiofrequency Ablation of Hepatocellular Carcinoma. OncoImmunology.

[95] Zerbini A., Pilli M., Fagnoni F., Pelosi G., Pizzi M.G., Schivazappa S., Laccabue D., Cavallo C., Schianchi C., Ferrari C. (2008). Increased Immunostimulatory Activity Conferred to Antigen-Presenting Cells by Exposure to Antigen Extract From Hepatocellular Carcinoma After Radiofrequency Thermal Ablation. J. Immunother..

[96] Zhang J., Sun Y., Li Y., Han J. (2024). Application of Combined Ablation and Immunotherapy in NSCLC and Liver Cancer: Current Status and Future Prospects. Heliyon.

[97] Qi X., Yang M., Ma L., Sauer M., Avella D., Kaifi J.T., Bryan J., Cheng K., Staveley-O’Carroll K.F., Kimchi E.T. (2020). Synergizing Sunitinib and Radiofrequency Ablation to Treat Hepatocellular Cancer by Triggering the Antitumor Immune Response. J. Immunother. Cancer.

[98] Gong Q., Qin Z., Hou F. (2017). Improved Treatment of Early Small Hepatocellular Carcinoma Using Sorafenib in Combination with Radiofrequency Ablation. Oncol. Lett..

[99] Fukuda H., Numata K., Moriya S., Shimoyama Y., Ishii T., Nozaki A., Kondo M., Morimoto M., Maeda S., Sakamaki K. (2014). Hepatocellular Carcinoma: Concomitant Sorafenib Promotes Necrosis after Radiofrequency Ablation—Propensity Score Matching Analysis. Radiology.

[100] Feng X., Xu R., Du X., Dou K., Qin X., Xu J., Jia W., Wang Z., Zhao H., Yang S. (2014). Combination therapy with sorafenib and radiofrequency ablation for BCLC Stage 0-B1 hepatocellular carcinoma: A multicenter retrospective cohort study. Am. J. Gastroenterol..

[101] Numata K., Wang F. (2022). New Developments in Ablation Therapy for Hepatocellular Carcinoma: Combination with Systemic Therapy and Radiotherapy. Hepatobiliary Surg. Nutr..

[102] Kan X., Jing Y., Wan Q.-Y., Pan J.-C., Han M., Yang Y., Zhu M., Wang Q., Liu K.-H. (2015). Sorafenib combined with percutaneous radiofrequency ablation for the treatment of medium-sized hepatocellular carcinoma. Eur. Rev. Med. Pharmacol. Sci..

[103] Wang F., Numata K., Komiyama S., Miwa H., Sugimori K., Ogushi K., Moriya S., Nozaki A., Chuma M., Ruan L. (2022). Combination Therapy With Lenvatinib and Radiofrequency Ablation for Patients With Intermediate-Stage Hepatocellular Carcinoma Beyond Up-To-Seven Criteria and Child--Pugh Class A Liver Function: A Pilot Study. Front. Oncol..

[104] Kudo M., Ueshima K., Nakahira S., Nishida N., Ida H., Minami Y., Nakai T., Wada H., Kubo S., Ohkawa K. (2022). Final Results of Adjuvant Nivolumab for Hepatocellular Carcinoma (HCC) after Surgical Resection (SR) or Radiofrequency Ablation (RFA) (NIVOLVE): A Phase 2 Prospective Multicenter Single-Arm Trial and Exploratory Biomarker Analysis. J. Clin. Oncol..

[105] Lyu N., Kong Y., Li X., Mu L., Deng H., Chen H., He M., Lai J., Li J., Tang H. (2020). Ablation Reboots the Response in Advanced Hepatocellular Carcinoma With Stable or Atypical Response During PD-1 Therapy: A Proof-of-Concept Study. Front. Oncol..

[106] Zhou C., Li Y., Li J., Song B., Li H., Liang B., Gu S., Li H., Chen C., Li S. (2023). A Phase 1/2 Multicenter Randomized Trial of Local Ablation plus Toripalimab versus Toripalimab Alone for Previously Treated Unresectable Hepatocellular Carcinoma. Clin. Cancer Res..

[107] Wang X., Liu G., Chen S., Bi H., Xia F., Feng K., Ma K., Ni B. (2021). Combination Therapy with PD-1 Blockade and Radiofrequency Ablation for Recurrent Hepatocellular Carcinoma: A Propensity Score Matching Analysis. Int. J. Hyperth..

[108] Wen Z., Wang J., Tu B., Liu Y., Yang Y., Hou L., Yang X., Liu X., Xie H. (2023). Radiofrequency Ablation Combined with Toripalimab for Recurrent Hepatocellular Carcinoma: A Prospective Controlled Trial. Cancer Med..

[109] Duffy A.G., Ulahannan S.V., Makorova-Rusher O., Rahma O., Wedemeyer H., Pratt D., Davis J.L., Hughes M.S., Heller T., ElGindi M. (2017). Tremelimumab in Combination with Ablation in Patients with Advanced Hepatocellular Carcinoma. J. Hepatol..

[110] Hiroishi K., Eguchi J., Baba T., Shimazaki T., Ishii S., Hiraide A., Sakaki M., Doi H., Uozumi S., Omori R. (2010). Strong CD8(+) T-Cell Responses against Tumor-Associated Antigens Prolong the Recurrence-Free Interval after Tumor Treatment in Patients with Hepatocellular Carcinoma. J. Gastroenterol..

[111] Ramsey D.E., Kernagis L.Y., Soulen M.C., Geschwind J.-F.H. (2002). Chemoembolization of Hepatocellular Carcinoma. J. Vasc. Interv. Radiol. JVIR.

[112] Li J., Zhang Y., Hu L., Ye H., Yan X., Li X., Li Y., Ye S., Wu B., Li Z. (2025). T-Cell Receptor Repertoire Analysis in the Context of Transarterial Chemoembolization Synergy with Systemic Therapy for Hepatocellular Carcinoma. J. Clin. Transl. Hepatol..

[113] Liu K., Min X.-L., Peng J., Yang K., Yang L., Zhang X.-M. (2016). The Changes of HIF-1α and VEGF Expression After TACE in Patients With Hepatocellular Carcinoma. J. Clin. Med. Res..

[114] Fu C., Chen H., Chen Y., Liu W., Cao G. (2024). Transarterial Intervention Therapy Combined with Systemic Therapy for HCC: A Review of Recent Five-Year Articles. Hepatoma Res..

[115] Kudo M., Imanaka K., Chida N., Nakachi K., Tak W.-Y., Takayama T., Yoon J.-H., Hori T., Kumada H., Hayashi N. (2011). Phase III study of sorafenib after transarterial chemoembolisation in Japanese and Korean patients with unresectable hepatocellular carcinoma. Eur. J. Cancer.

[116] Kudo M., Ueshima K., Ikeda M., Torimura T., Tanabe N., Aikata H., Izumi N., Yamasaki T., Nojiri S., Hino K. (2022). Final Results of TACTICS: A Randomized, Prospective Trial Comparing Transarterial Chemoembolization Plus Sorafenib to Transarterial Chemoembolization Alone in Patients with Unresectable Hepatocellular Carcinoma. Liver Cancer.

[117] Meyer T., Fox R., Ma Y.T., Ross P.J., James M.W., Sturgess R., Stubbs C., Stocken D.D., Wall L., Watkinson A. (2017). Sorafenib in combination with transarterial chemoembolisation in patients with unresectable hepatocellular carcinoma (TACE 2): A randomised placebo-controlled, double-blind, phase 3 trial. Lancet Gastroenterol. Hepatol..

[118] Lencioni R., Llovet J.M., Han G., Tak W.Y., Yang J., Guglielmi A., Paik S.W., Reig M., Kim D.Y., Chau G.-Y. (2016). Sorafenib or placebo plus TACE with doxorubicin-eluting beads for intermediate stage HCC: The SPACE trial. J. Hepatol..

[119] Kudo M., Arizumi T. (2017). Transarterial Chemoembolization in Combination with a Molecular Targeted Agent: Lessons Learned from Negative Trials (Post-TACE, BRISK-TA, SPACE, ORIENTAL, and TACE-2). Oncology.

[120] Kudo M., Ueshima K., Saeki I., Ishikawa T., Inaba Y., Morimoto N., Aikata H., Tanabe N., Wada Y., Kondo Y. (2024). A Phase 2, Prospective, Multicenter, Single-Arm Trial of Transarterial Chemoembolization Therapy in Combination Strategy with Lenvatinib in Patients with Unresectable Intermediate-Stage Hepatocellular Carcinoma: TACTICS-L Trial. Liver Cancer.

[121] Peng Z., Fan W., Zhu B., Wang G., Sun J., Xiao C., Huang F., Tang R., Cheng Y., Huang Z. (2023). Lenvatinib Combined With Transarterial Chemoembolization as First-Line Treatment for Advanced Hepatocellular Carcinoma: A Phase III, Randomized Clinical Trial (LAUNCH). J. Clin. Oncol..

[122] (2021). IMMUTACE IMMUTACE: A Phase 2 Single-Arm, Open-Label Study of Transarterial Chemoembolization in Combination With Nivolumab Performed for Intermediate-Stage Hepatocellular Carcinoma. Gastroenterol. Hepatol..

[123] Pinato D.J., D’Alessio A., Fulgenzi C.A.M., Schlaak A.E., Celsa C., Killmer S., Blanco J.M., Ward C., Stikas C.-V., Openshaw M.R. (2024). Safety and Preliminary Efficacy of Pembrolizumab Following Transarterial Chemoembolization for Hepatocellular Carcinoma: The PETAL Phase Ib Study. Clin. Cancer Res..

[124] Guo C., Zhang J., Huang X., Chen Y., Sheng J., Huang X., Sun J., Xiao W., Sun K., Gao S. (2023). Preoperative sintilimab plus transarterial chemoembolization for hepatocellular carcinoma exceeding the Milan criteria: A phase II trial. Hepatol. Commun..

[125] Sangro B., Harding J.J., Johnson M., Palmer D.H., Edeline J., Abou-Alfa G.K., Cheng A.-L., Decaens T., El-Khoueiry A.B., Finn R.S. (2021). A phase III, double-blind, randomized study of nivolumab (NIVO) and ipilimumab (IPI), nivo monotherapy or placebo plus transarterial chemoembolization (TACE) in patients with intermediate-stage hepatocellular carcinoma (HCC). J. Clin. Oncol..

[126] Sangro B., Kudo M., Erinjeri J.P., Qin S., Ren Z., Chan S.L., Arai Y., Heo J., Mai A., Escobar J. (2025). Durvalumab with or without bevacizumab with transarterial chemoembolisation in hepatocellular carcinoma (EMERALD-1): A multiregional, randomised, double-blind, placebo-controlled, phase 3 study. Lancet.

[127] Du J.-S., Hsu S.-H., Wang S.-N. (2024). The Current and Prospective Adjuvant Therapies for Hepatocellular Carcinoma. Cancers.

[128] Patel T.H., Brewer J.R., Fan J., Cheng J., Shen Y.-L., Xiang Y., Zhao H., Lemery S.J., Pazdur R., Kluetz P.G. (2024). FDA Approval Summary: Tremelimumab in Combination with Durvalumab for the Treatment of Patients with Unresectable Hepatocellular Carcinoma. Clin. Cancer Res. Off. J. Am. Assoc. Cancer Res..

[129] Kudo M., Ren Z., Guo Y., Han G., Lin H., Zheng J., Ogasawara S., Kim J.H., Zhao H., Li C. (2025). Transarterial chemoembolisation combined with lenvatinib plus pembrolizumab versus dual placebo for unresectable, non-metastatic hepatocellular carcinoma (LEAP-012): A multicentre, randomised, double-blind, phase 3 study. Lancet.

[130] Vilgrain V., Pereira H., Assenat E., Guiu B., Ilonca A.D., Pageaux G.-P., Sibert A., Bouattour M., Lebtahi R., Allaham W. (2017). Efficacy and safety of selective internal radiotherapy with yttrium-90 resin microspheres compared with sorafenib in locally advanced and inoperable hepatocellular carcinoma (SARAH): An open-label randomised controlled phase 3 trial. Lancet Oncol..

[131] Chow P.K.H., Gandhi M., Tan S.-B., Khin M.W., Khasbazar A., Ong J., Choo S.P., Cheow P.C., Chotipanich C., Lim K. (2018). SIRveNIB: Selective Internal Radiation Therapy Versus Sorafenib in Asia-Pacific Patients With Hepatocellular Carcinoma. JCO.

[132] Seidensticker M., Öcal O., Schütte K., Malfertheiner P., Berg T., Loewe C., Klümpen H.J., van Delden O., Ümütlü M.R., Ben Khaled N. (2023). Impact of adjuvant sorafenib treatment after local ablation for HCC in the phase II SORAMIC trial. JHEP Rep..

[133] Fenton S.E., Kircher S.M., Mulcahy M.F., Mahalingam D., Salem R., Lewandowski R., Kulik L., Benson A.B., Kalyan A. (2021). A Phase I Study of Nivolumab (NIVO) in Combination with TheraSphere (Yttrium-90) in Patients with Advanced Hepatocellular Cancer. J. Clin. Oncol..

[134] de la Torre-Aláez M., Matilla A., Varela M., Iñarrairaegui M., Reig M., Lledó J.L., Arenas J.I., Lorente S., Testillano M., Márquez L. (2022). Nivolumab after selective internal radiation therapy for the treatment of hepatocellular carcinoma: A phase 2, single-arm study. J. Immunother. Cancer.

[135] Yu S., Yu M., Keane B., Mauro D.M., Helft P.R., Harris W.P., Sanoff H.K., Johnson M.S., O’Neil B., McRee A.J. (2024). A Pilot Study of Pembrolizumab in Combination With Y90 Radioembolization in Subjects With Poor Prognosis Hepatocellular Carcinoma. Oncol..

[136] Llovet J.M., Vogel A., Madoff D.C., Finn R.S., Ogasawara S., Ren Z., Mody K., Li J.J., Siegel A.B., Dubrovsky L. (2022). Randomized Phase 3 LEAP-012 Study: Transarterial Chemoembolization With or Without Lenvatinib Plus Pembrolizumab for Intermediate-Stage Hepatocellular Carcinoma Not Amenable to Curative Treatment. Cardiovasc. Intervent. Radiol..

